# Antiplasmodial Natural Products

**DOI:** 10.3390/molecules16032146

**Published:** 2011-03-04

**Authors:** Cláudio R. Nogueira, Lucia M. X. Lopes

**Affiliations:** Instituto de Química, Universidade Estadual Paulista, UNESP, C. P. 355, 14801-970, Araraquara, SP, Brazil; E-Mail: nogueiracr@rocketmail.com (C.R.N.)

**Keywords:** malaria, antimalarial, antiplasmodial, *Plasmodium*

## Abstract

Malaria is a human infectious disease that is caused by four species of *Plasmodium.* It is responsible for more than 1 million deaths per year. Natural products contain a great variety of chemical structures and have been screened for antiplasmodial activity as potential sources of new antimalarial drugs. This review highlights studies on natural products with antimalarial and antiplasmodial activity reported in the literature from January 2009 to November 2010. A total of 360 antiplasmodial natural products comprised of terpenes, including iridoids, sesquiterpenes, diterpenes, terpenoid benzoquinones, steroids, quassinoids, limonoids, curcubitacins, and lanostanes; flavonoids; alkaloids; peptides; phenylalkanoids; xanthones; naphthopyrones; polyketides, including halenaquinones, peroxides, polyacetylenes, and resorcylic acids; depsidones; benzophenones; macrolides; and miscellaneous compounds, including halogenated compounds and chromenes are listed in this review.

## 1. Introduction

Malaria is an infectious disease caused by four protozoan species of the genus *Plasmodium* (*Plasmodium falciparum*, *Plasmodium malariae*, *Plasmodium ovale,* and *Plasmodium vivax*) [1]. Small human infection outbreaks caused by a malaria parasite of monkeys, *Plasmodium knowlesi*, have also been reported in Southeast Asia [2]. The majority of the cases of malaria and deaths (malaria kills 1-2 million people each year) are caused by *P. falciparum. P. vivax* is generally considered less dangerous than *P. falciparum*, although both can cause deadly complications in infected people. Nearly 3 billion people are at risk of infection with the malaria parasite *P. vivax.* A new map of areas where this parasite has been reported, including risk areas, has been drawn [3]. These grim statistics could become even worse if resistance to the existing antimalarial drugs develops further [4]. According to WHO [5], the elimination of malaria from countries with high transmission rates is a long-term goal that will depend on the success of research and development to deliver a more robust arsenal of tools than those available today – tools of greater potency and effectiveness, especially those with an impact on transmission, and replacements for medicines and insecticides that are being lost to resistance. The growing resistance to existing antimalarial drugs could nullify efforts to eliminate this deadly disease. Unfortunately, there are still very few drugs that are active against malaria (artemisinin, atovaquone, and chloroquine analogues) [4,6] and vaccines against malaria are not yet available [6]. 

At present, drug resistance of the malaria parasite is widespread, no new chemical class of antimalarials has been introduced into clinical practice since 1996, and there has recently been an increase in parasite strains with reduced sensitivity to the newest drugs [7]. Meanwhile, recent advances in genome-based technologies and *in vitro* screening of whole parasites and a great number of compounds (natural and synthetic) have broadened the range of therapeutic targets and are accelerating the development of a new generation of treatments for both the control and eradication of malaria [6].

Several excellent reviews on antiplasmodial compounds, including natural products, have been published in recent years [8,9,10,11,12,13,14,15,16,17,18]. Thousands of chemicals have been assayed for antiplasmodial activity, such as those described by Gamo et al. [7]. 

The current review provides an overview of a great number of bioactive natural products that have recently been described in the literature (from January 2009 to November 2010) as showing antiplasmodial activity (*in vitro*), along with a few compounds that were tested for antimalarial activity in animal models [19] using *Plasmodium knowlesi* (in simians), *Plasmodium yoelii*, *Plasmodium berghei, Plasmodium chabaudi* (in mice), and *Plasmodium gallinaceum* (in birds). In most of these bioassays, antiplasmodial activities were assessed using different *P. falciparum* strains, which include chloroquine-sensitive (NF54, NF54/64, 3D7, D6, F32, D10, HB3, FCC1-HN, Ghana, MRC-02, TM4), chloroquine-resistant (BHz26/86, Dd2, EN36, ENT30, FcB1, FCM29, FCR3, FCR-3/A2, FCR3F86, S20, W2,), chloroquine-resistant and pyrimethamine-resistant (K1, TM91C235), pyrimethamine-resistant (HB3), cycloguanil-resistant (CDC1), and chloroquine- and antifolate-resistant (K1CB1). Most of evaluations used the [^3^H]-hypoxanthine-incorporation assay to assess parasite inhibition of growth in the presence of the test-drugs. Antimalarial activity of new compounds has also been determined by using: i) the fluorometric method based on the intercalation of the fluorochrome PicoGreen (SYBR) in the parasite DNA, [20]; ii) enzyme-linked immunosorbent assays (ELISAs) with monoclonal antibodies, which measure the *P. falciparum*-specific antigen histidine-rich protein 2 (HRP2) or lactate dehydrogenase protein (pLDH). A chemical reaction using ferriprotoporphyrine biocrystallization (FBTI Inhibition Test) has been used to provide a possible action mechanism for presumed antimalarial compounds [21]. Protein farnesyltransferase (FTase) bioassays have also been used to provide insight into their mode of action against *P. falciparum* [22]. The effects of natural products on glutathione (GSH), which plays a key role in redox mechanisms, and on cysteine (Cys), which is one of the substrates needed for the de novo synthesis of *P. falciparum* GSH, as well as their impact on β-hematin formation have been investigated, since GSH participates in heme detoxification [23]. The advantages and disadvantages of the different *in vitro* screening methods have been discussed by Krettli et al. [24] and Wein et al. [25]. For *in vivo* bioassays *P. berghei* and *P. chabaudi chabaudi* have been used most often. The cytotoxicities of the active compounds compiled in this review have generally been evaluated in HEK293, Vero or HeLa cells. For the details of the methodologies, see the appropriate references cited herein. 

Several criteria have been proposed for considering a compound as active. Generally, a compound is considered to be inactive when it shows an IC_50_ > 200 μM, whereas those with an IC_50_ of 100-200 μM have low activity; IC_50_ of 20-100 μM, moderate activity; IC_50_ of 1-20 μM good activity; and IC_50_ < 1 μM excellent/potent antiplasmodial activity [12]. In this review, regardless of the *in vitro* or *in vivo* method adopted for antiplasmodial or antimalarial evaluation, we list the active and moderately active compounds in accordance with the corresponding cited literature (data for inactive compounds are not shown in this review).

A total of 360 antiplasmodial natural products comprised of terpenes, including iridoids, sesquiterpenes, diterpenes, terpenoid benzoquinones, steroids, quassinoids, limonoids, curcubitacins, and lanostanes; flavonoids; alkaloids; peptides; phenylalkanoids; xanthones; naphthopyrones; polyketides, including halenaquinones, peroxides, polyacetylenes, and resorcylic acids; depsidones; benzophenones; macrolides; and miscellaneous compounds, including halogenated compounds and chromenes are listed in this compilation (Figures 1-44).

## 2. Terpenes

### 2.1. Iridoids and halogenated monoterpenes

**Figure 1:**
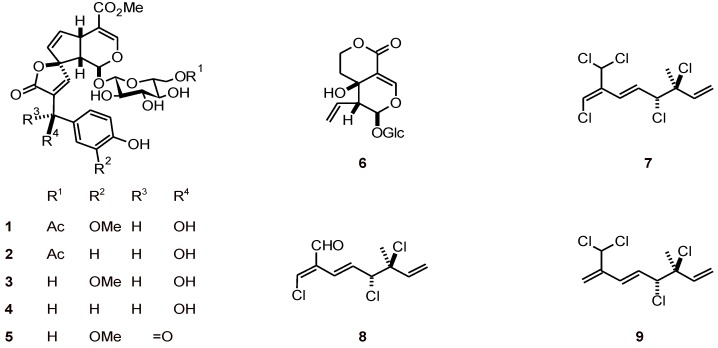
Structures of iridoids and halogenated monoterpenes **1**-**9**.

Phenylpropanoid conjugated iridoids **1**-**5** (Figure 1) have been isolated from *Morinda morindoides* (Rubiaceae)*.* All of these compounds except for **3** potently inhibited parasite proliferation (*P. falciparum* strain CDC1, IC_50_ values of 0.04 to 4.1 µM) with little cytotoxicity against the host mammalian cells (KB 3-1) [26]. The iridoid swertiamarin (**6**) has been obtained from *Enicostemma littorale* (Gentianaceae) and showed promising results *in vitro* in a schizont maturation inhibition assay, with an IC_50_ value of 44.4 µΜ [27]. 

Halogenated monoterpenes **7**-**9** with a 3,7-dimethyl-3,4-dichloro-octa-1,5,7-triene skeleton obtained from the marine red alga *Plocamium cornutum* (Plocamiaceae) exhibited antiplasmodial activity toward a chloroquine-sensitive strain of *P. falciparum*. Those bearing a 7-dichloromethyl substituent showed higher activity, with IC_50_ values ranging from 16 to 27 μM [28].

### 2.2. Sesquiterpenes

**Figure 2:**
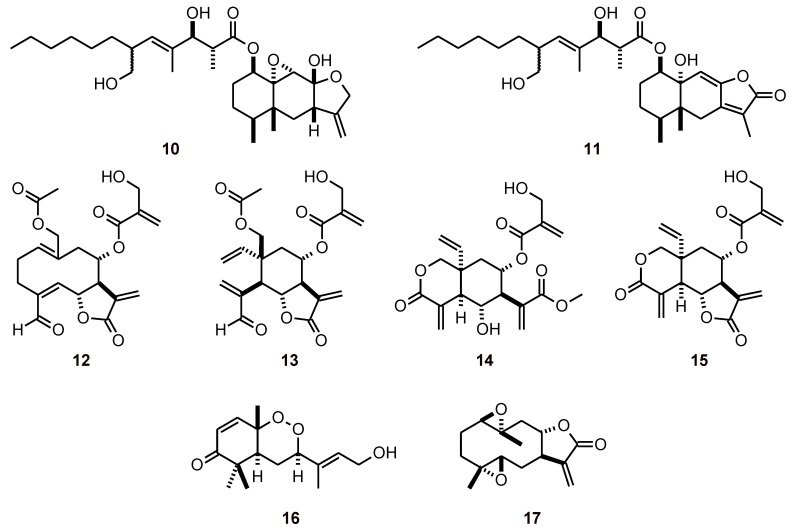
Structures of sesquiterpenes **10-17**.

The eremophilane sesquiterpenoids berkleasmins A (**10**) and C (**11**) (Figure 2), obtained from the saprobic fungus *Berkleasminum nigroapicale*, showed antiplasmodial activity with IC_50_ values of 6.0 and 5.4 µΜ, respectively [29]. The sesquiterpene lactones vernaguline A and B, vernodalol, and vernodalin (**12-15**) were isolated from *Distephanus angulifolius* (Asteraceae), and while they exhibited antiplasmodial activity against chloroquine-sensitive (D10) and chloroquine-resistant (W2) *P. falciparum* strains (IC_50_ values of 1.5 to 4.9 μM), they also exhibited cytotoxicity in a hamster ovarian cell line [30].

Okundoperoxide (**16**) was isolated by the bioassay-guided fractionation of extracts from *Scleria striatinux* (syn. *S. striatonux*, Cyperaceae). This compound contains a cyclic endoperoxide structural moiety and showed antiplasmodial activity against W2 and D6 (IC_50_ ~1.8 µΜ), K1 (IC_50_ = 5.6 µΜ), and NF54 (IC_50_ = 4.9 µΜ) [31].

Studies on the *Carpesium* genus (Compositae) suggest that the antiplasmodial activity against *P. falciparum* is due to the presence of 11(13)-dehydroivaxillin (**17**) in the EtOAc extracts of *C. cernuum*. The antimalarial activity of **17** was evaluated against *P. berghei* in mice. Its LD_50_ was determined to be 51.2 mg/kg, while doses of 124 mg/kg and above were found to be lethal to mice. DDV (2, 5, 10 mg/kg/day) exhibited a significant blood schizonticidal activity in 4-day early infection, repository evaluation and in an established infection with a significant mean survival time comparable to that of the standard drug (chloroquine, 5 mg/kg/day) [32].

**Figure 3:**
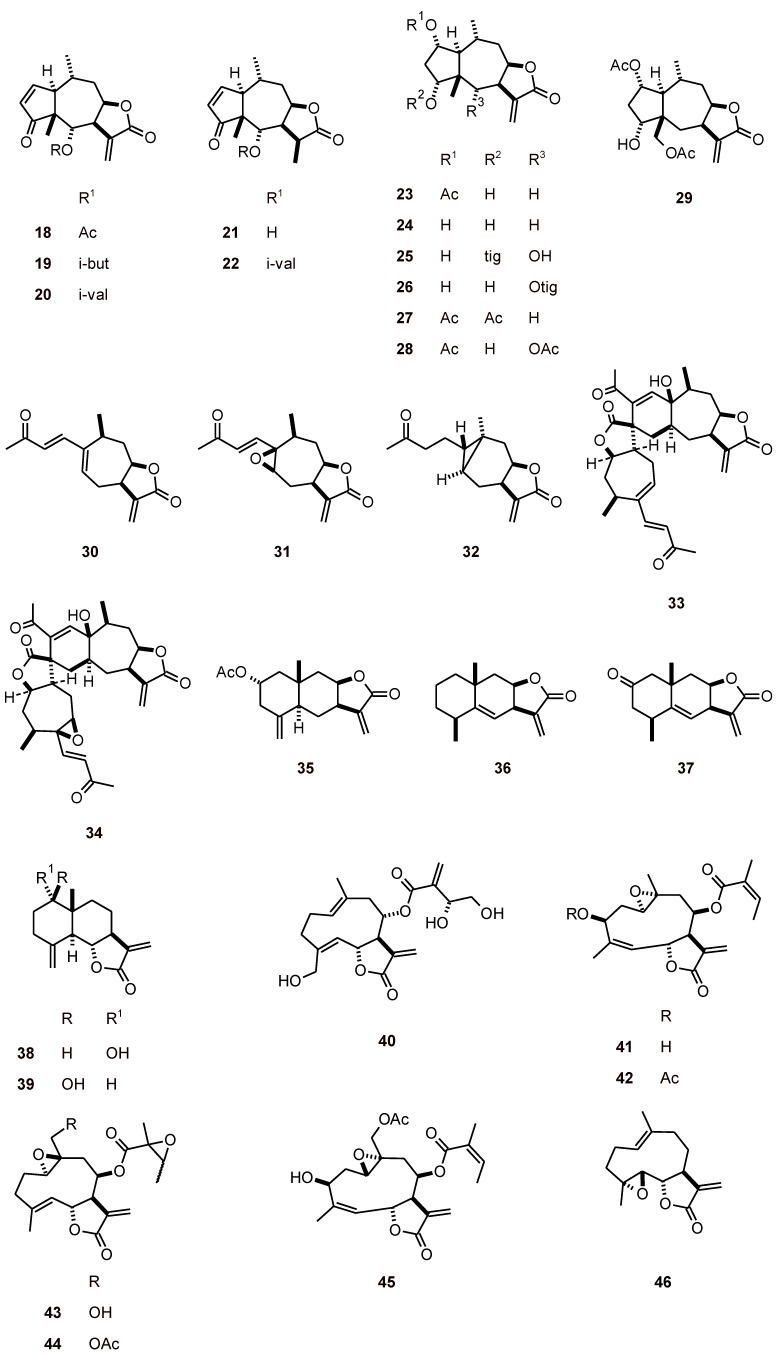
Structures of sesquiterpenes **18-46**.

In tests of 33 sesquiterpene lactones from several genera, including *Arnica*, *Xanthium*, and *Inula* (Asteraceae), Schmidt *et al*. [33] found that the antiprotozoal activities were significantly correlated with cytotoxicity, and the major determinants of this activity were α,β-unsaturated structural elements. Among the tested sesquiterpene lactones, 29 (compounds **18-46**, Figure 3) showed activity against *P. falciparum* (K1) with IC_50_ values of 0.3 to 27.5 µM [33]. 

### 2.3. Diterpenes

**Figure 4:**
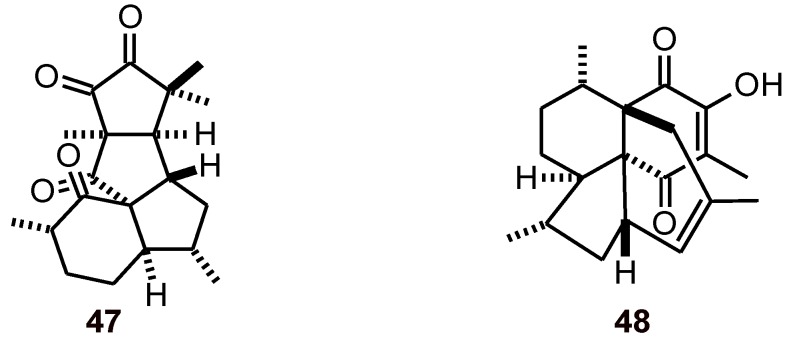
Structures of diterpenes **47** and **48**.

Aberrarone A (**47**, Figure 4) is a natural product from the Caribbean sea whip *Pseudopterogorgia elisabethae* (Gorgoniidae)*.* This compound, together with colombiasin A (**48**), showed *in vitro* activity against a chloroquine-resistant strain of *P. falciparum* (W2, IC_50_ values of 30.3 and 31.8 μM, respectively) with the use of a fluorometric method (PicoGreen) [34]. 

**Figure 5:**
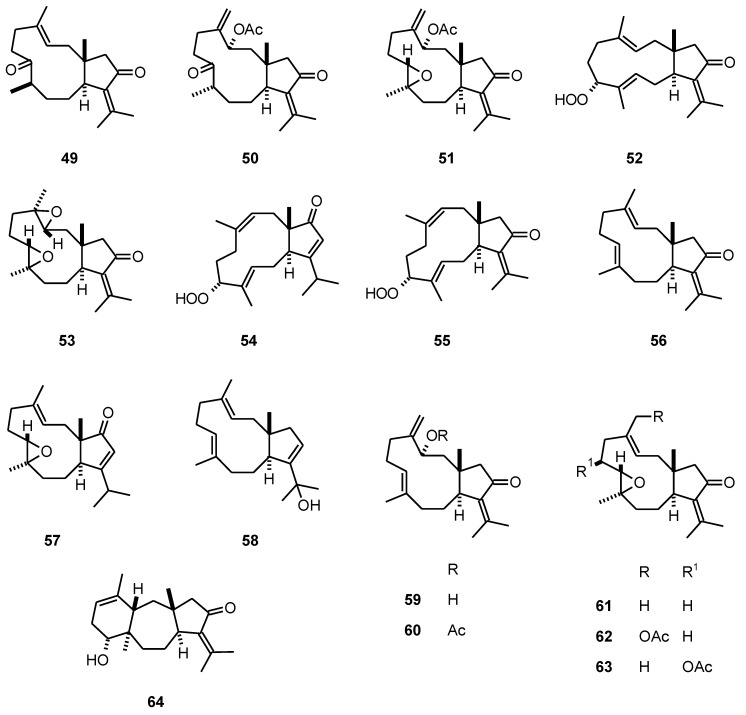
Structures of dolabellanes **49**-**63**.

The dolabellanes **49-63 **(Figure 5) and dolastane **64 **from a *Eunicea* species Caribbean octocoral (Gorgoniidae) were tested for their inhibitory activity toward the growth of *P. falciparum* (W2). These compounds were active with IC_50_ values of 9.4 to 59.6 μM [35].

**Figure 6:**
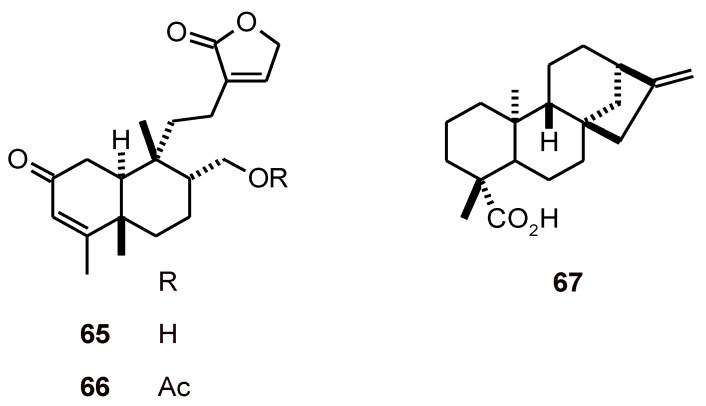
Structures of clerodane and kaurane diterpenes **65-67**.

Gomphostenin clerodane diterpenes **65** and **66** (Figure 6) obtained from *Gomphostemma niveum* (Lamiaceae) were tested against *P. berghei* in mice and produced a dose-dependent chemo-suppression effect. Diterpene **65** exhibited the highest percent of chemo-suppression, i.e. 92.65% at a dose of 200 mg/kg/day. In a curative test, the survival period of the infected mice was significantly prolonged at 200 mg/kg dose of 66 [36]. Gomphostenin (**65**) and Gomphostenin-A (**66**) exhibited *in vitro* antiplasmodial activities against the MRC-02 strain of *P. falciparum* with IC_50_ values of 114.9 and 9.8 µM, respectively [37]. In contrast, the antiplasmodial activity of *ent*-kaur-16-en-19-oic acid (**67**) from *Schefflera umbellifera* (Araliaceae) against the chloroquine-susceptible strain D10 (IC_50_ = 106.5 µM) was not very significant [38].

### 2.4. Nitrogenated diterpenes

**Figure 7:**
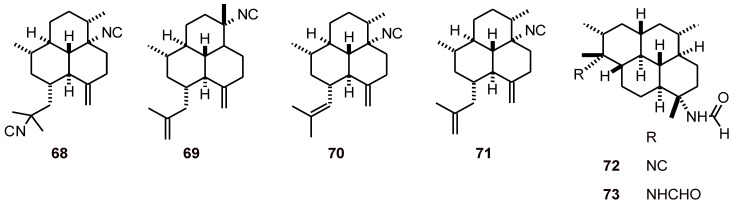
Structures of nitrogenated diterpenes **68**-**73**.

Isonitrile diterpenes **68**-**71** (Figure 7) of the amphilectane family have been isolated from the sponge *Ciocalapata* sp. (Halichondriidae). They exhibited strong activities against *P. falciparum* (K1) with IC_50_ values of 0.09 to 1.07 µM. Except for 8,15-diisocyano-11(20)-amphilectene (**68**), which was cytotoxic against both MCF-7 and fibroblast cell lines, these diterpenes showed no significant cytotoxicity against either of the targeted cell lines [39]. Alkaloid **72** is also a formamide and isonitrile, diterpene that was isolated from the tropical marine sponge *Cymbastela hooperi* (Axinellidae)*.* In *in vitro* antiplasmodial bioassays using three strains (FCR3F86, W2, and D6) of the malaria parasite *P. falciparum*, **72** was found to have good activity (IC_50_ = 1.5 µM), whereas the corresponding di-formamide derivative **73** showed only moderate activity (IC_50_ = 41.0 µM) [40].

### 2.5. Tetranorditerpenes

**Figure 8:**
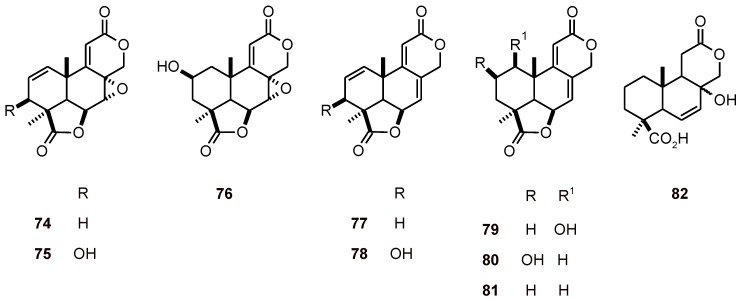
Structures of tetranorditerpenes 74-82.

Tetranorditerpenoid dilactones **74**-**81** and an oidiolactone **82** (Figure 8), isolated from the fungus *Sclerotinia homoeocarpa,* exhibited excellent to good antiplasmodial activity (IC_50_ values of 0.1 to 8.0 µM). However, they also showed high cytotoxicity, which preclude their use as potential antimalarial agents [41].

### 2.6. Terpenoid benzoquinones and analogues

**Figure 9:**
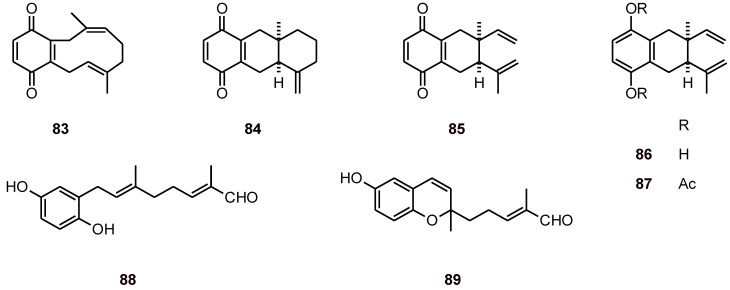
Structures of terpenoid benzoquinones and analogues 83-89.

Terpenoid benzoquinones and analogues **83-89** (Figure 9) isolated from the root extract of *Cordia globifera* (Boraginaceae) exhibited antiplasmodial activity against the *P. falciparum* strain K1 with IC_50_ values of 0.8 to 13.9 μM. The antifungal and cytotoxic activities of these compounds were also evaluated [42].

### 2.7. Steroids

**Figure 10:**
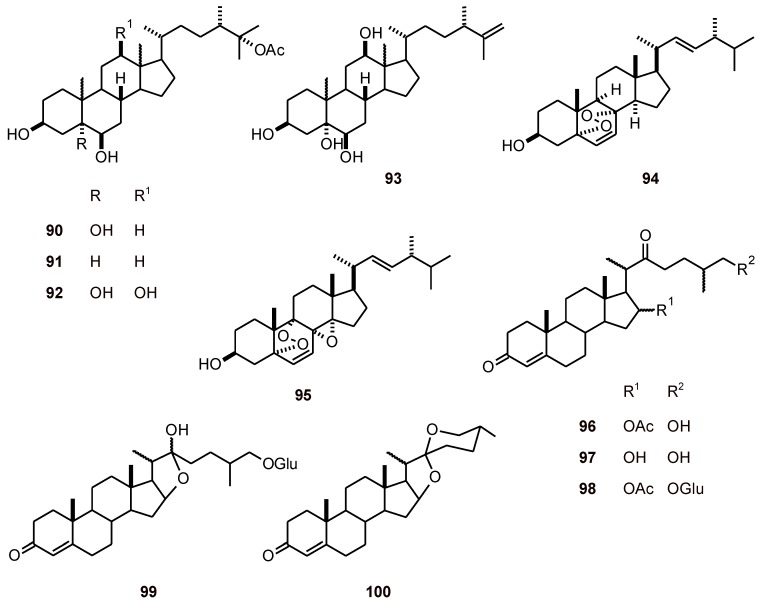
Structures of steroids **90**-**100**.

The major steroid **90** together with three other steroids **91**-**93** (Figure 10) isolated from the marine sponge *Callyspongia fibrosa* (Callyspongiidae) showed antiplasmodial activity (*P. falciparum* strains 3D7 and K1 with IC_50_ values of 20.5 to 54.8 µM). Parasite growth was assessed as pLDH activity and **90** exhibited better activity against a chloroquine-resistant strain of *P. falciparum* than on a chloroquine-sensitive strain [43]. Two steroidal peroxides **94 **and **95** from another marine sponge *Ciocalapata sp*. (Halichondriidae) showed antiplasmodial activity against *P. falciparum* K1 (IC_50 _values of 6.28 and 7.13 µM, respectively), and cytotoxicity against human cells in a breast cancer cell line MCF-7 (IC_50_ values of 0.025 and 0.003 µM, respectively), with very little toxicity against human fibroblasts [39].

An evaluation of the effects of four steroid derivatives **96-99** and a sapogenin **100** extracted from *Solanum nudum* (Solanaceae) on the total glutathione (GSH) and cysteine contents in *P. falciparum in vitro* showed that **96** increased total glutathione and cysteine concentrations while **99** decreased the concentrations of both thiols. Acetylation at C16 was crucial for the effect of **96** while type furostanol and terminal glucosidation were necessary for the inhibitory properties of **99**. The combination of steroids and buthionine sulfoximine, a specific inhibitor of a step-limiting enzyme in GSH synthesis, did not modify the glutathione contents. In addition, **96** inhibited more than 80% of β-hematin formation at 5.0 mM, while the other steroids did not show any effect [23].

### 2.8. Quassinoids

**Figure 11:**
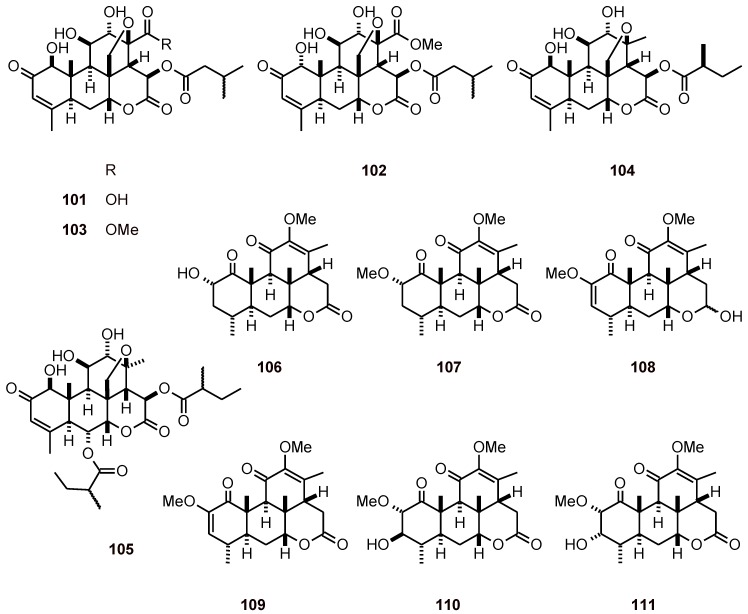
Structures of quassinoids **101**-**111**.

Delaumonones A (**101**) and B (**102**) (Figure 11) have been isolated from the bark of *Laumoniera bruceadelpha* (Simaroubaceae)*.* These quassinoids showed antiplasmodial activity against the *P. falciparum* strain 3D7 (IC_50_ values of 0.6 and 1.2 µM, respectively) and cytotoxicity against HL-60 cells (IC_50_ **101**: 3.1 µM; **102**: 4.6 µM). Another isolated quassinoid, the isobrucein A (**103**) (IC_50_ = 0.05 µM), was more active than delaumonones A and B, but it also showed a potent cytotoxicity against HL-60 cells (IC_50_**=** 0.01 µM) [44]. The tea of young leaf of *Quassia amara* (Simaroubaceae) also contains several quassinoids **104**-**111**, including simalikalactone D (**104**) and simalikalactone E (**105**) [45,46]. These quassinoids inhibited the growth of *P. falciparum* cultured *in vitro* (with IC_50_ values of 1 to 420 nM), independently of the strain sensitivity to chloroquine. Compound **105** decreased gametocytemia with an IC_50_ value seven-fold lower than that of primaquine, and was also less toxic than simalikalactone D (**104**) when tested on nontumorogenic cells. *In vivo*, **105** inhibited murine malaria growth of *P. vinckei petteri* by 50% at 1 and 0.5 mg/kg of body weight/day, by the oral or intraperitoneal routes, respectively [45,46].

### 2.9. Limonoids

**Figure 12:**
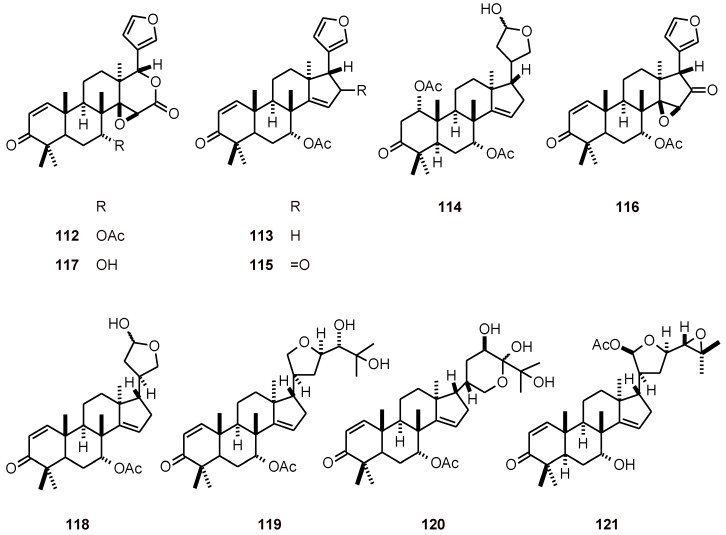
Structures of limonoids **112-121.**

*In vitro* antiplasmodial tests using the D10 and W2 strains of *P. falciparum* showed that gedunin (**112**), azadirone (**113**), and neemfruitin A (**114**) had significant activity, and limonoids **115**-**121** from *Azadirachta indica* (Meliaceae) had a good activity (IC_50_ values of 1.2 to 10.0 µM) (Figure 12) [47].

**Figure 13:**
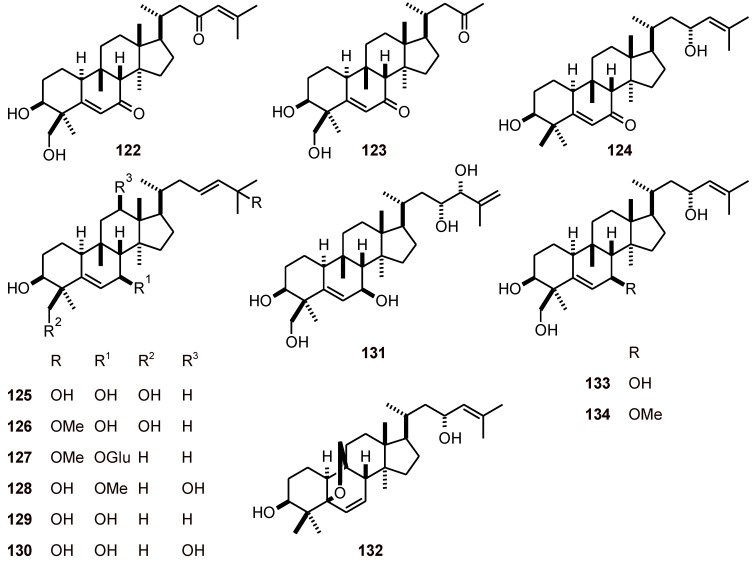
Structures of cucurbitacins **122-134.**

### 2.10. Cucurbitacins

Thirteen cucurbitane-type triterpenes **122**-**134** (Figure 13) have been isolated from *Momordica balsamina* (Curcubitaceae) and have been shown to exhibit antiplasmodial activity against the *P. falciparum* chloroquine-sensitive strain 3D7 and the chloroquine-resistant strain Dd2. Triterpenes **130** and **133** had the highest antiplasmodial effects against both strains (IC_50_**130**: 4.6 and **133**: 7.4 μM, 3D7; **130**: 4.0 and **133**: 8.2 μM, Dd2). Furthermore, a preliminary evaluation of the toxicity of compounds **122**-**126** and **130** toward human cells in a breast cancer cell line (MCF-7) showed that these triterpenes were inactive or showed only weak toxicity (IC_50_ values >19.0 μM) [48].

### 2.11. Lanostanes

**Figure 14:**
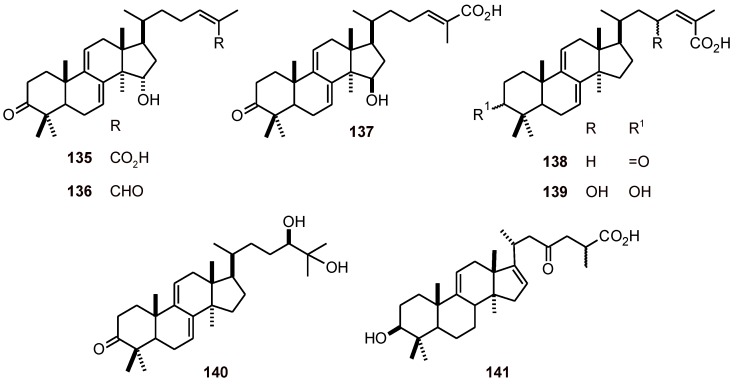
Structures of lanostanes **135**-**141**.

Ethyl acetate extract of the mushroom *Ganoderma lucidum* (Polyporaceae) yielded six lanostanes **135**-**140** (Figure 14). These lanostanes exhibited *in vitro* antiplasmodial activity with IC_50_ values of 6 to 20 *μ*M [49]. Another lanostane, garcihombronane D (**141**), from *Garcinia cymosa* (Clusiaceae) showed a selective activity against *P. falciparum* (IC_50_ 7.7 µM) [50].

### 2.12. Other triterpenes

**Figure 15:**
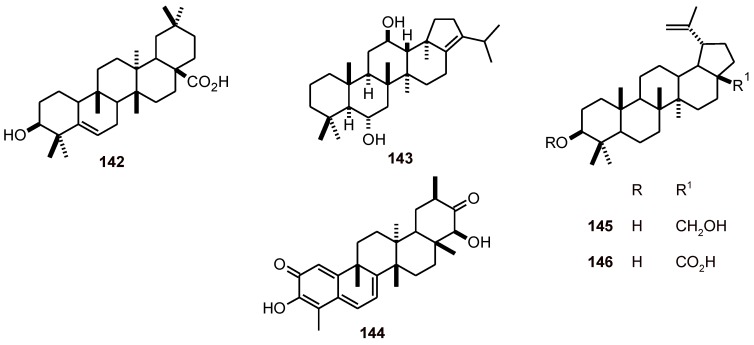
Structures of terpenes **142**-**146**.

The triterpene 3β-hydroxy-glutin-5-ene, which was also isolated from *Garcinia cymosa* (**142**) (Figure 15), showed activity against *P. falciparum* (IC_50_ 31.0 µM) and a cytotoxicity of 6.9 µM toward MRC-5 cells [50]. Hop-17(21)-en-6*R*,12-diol (**143**) was isolated from the scale insect pathogenic fungus *Aschersonia paraphysata*. It exhibited antiplasmodial activity with an IC_50_ value of 15 μM [51]. Tingenin B, (22β- hydroxytingenone) (**144**), has been isolated as the main antibacterial constituent from *Elaeodendron schlechteranum* (Celastraceae). It was active against *T. cruzi* (IC_50_ < 0.6 μM), *T. brucei* (IC_50_ < 0.6 μM), *L. infantum* (IC_50_ = 1.2 μM), and *P. falciparum* (IC_50_ = 0.8 μM). Tingenin B was highly cytotoxic to MRC-5 cells (CC_50_ 1.0 μM), which indicates poor selectivity [52]. Betulin (**145**) has been isolated from *Schefflera umbellifera* (Araliaceae), and exhibited good antiplasmodial activity (IC_50_ = 7.2 μM) against a chloroquine-susceptible strain (D10) [38]. Betulinic acid (**146**) showed antiplasmodial activity against chloroquine-resistant *P. falciparum* parasites (W2 strain, chloroquine-resistant and mefloquine sensitive) *in vitro*, with an IC_50_ value of 9.9 μM [53]*.*

## 3. Flavonoids

**Figure 16:**
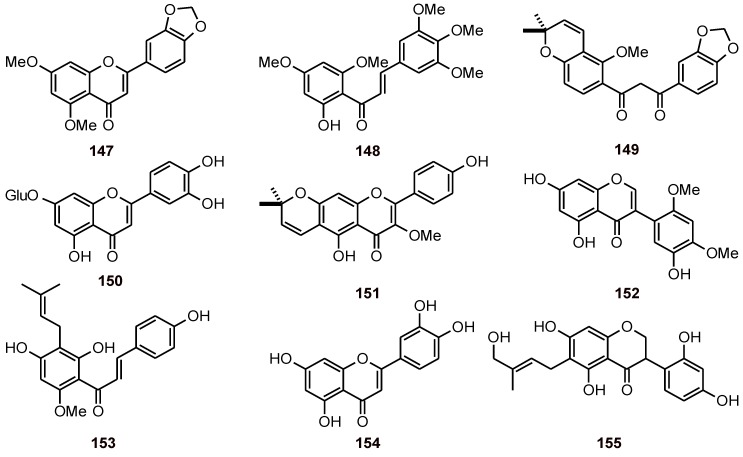
Structures of flavonoids **147-155**.

Flavonoids 147 and 148 have been isolated from *Neoraputia magnifica* (Rutaceae). These flavonoids, together with 149 (Figure 16), isolated from *Lonchocarpus subglaucescens* (Leguminosae), exhibited antiplasmodial activity against the *P. falciparum* strain 3D7 with IC_50_ values of 7.6, 6.9, and 9.5 µM, respectively [54]. The luteolin 7-*O*-β-D-glucopyranoside 150 also showed antiplasmodial activity (IC_50_ = 4.9 μM for the D6 strain and 4.0 μM for the W2 strain), and this compound did not exhibit cytotoxicity in Vero cells up to a concentration of 10.6 μM [55].

The flavone 3-methoxycarpachromene (**151**) isolated from *Pistacia atlantica* (Anacardiaceae) showed an IC_50_ value of 3.4 µM toward the *P. falciparum* strain K1 [56], whereas dalparvone (**152**) from the stems of *Dalbergia parviflora* (Leguminosae) exhibited moderate antimalarial activity with an IC_50_ value of 24.8 μM [57].

The *in vitro* antiplasmodial activities of the main hop chalcone xanthohumol (**153**) and seven of its derivatives were evaluated against two strains of *P. falciparum* (poW, Dd2). Xanthohumol had the highest activity, with IC_50_ values of 8.2 (poW) and 24.0 μM (Dd2) [58]. 

Compounds obtained from *Morinda morindoides* (Rubiaceae) were evaluated *in vitro* for antiplasmodial activity against a Congolese chloroquine-sensitive strain of *P. falciparum*. Quercetin (**154**) exhibited good antiplasmodial activity with an IC_50_ value of 19.2 μΜ, whereas alizarin and chrysazin displayed only moderate activity (58.3 < IC_50_ < 124.9 μM) [59]. 

The isoflavanone **155** has been isolated from *Ormocarpum kirkii* (Papilionaceae) and has been shown to have antiplasmodial activity with an IC_50_ value of 23.7 μM [60].

**Figure 17:**
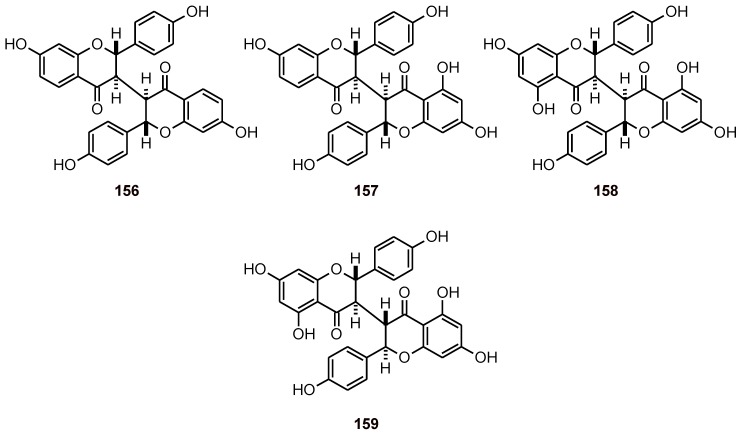
Structures of biflavonoids **156**-**159**.

*Ormocarpum kirkii* also gave four biflavonoids **156**-**159** (Figure 17) that showed antiplasmodial activity toward *P. falciparum* strain K1; isochamaejasmin (**159**) was the most active, with an IC_50_ of 7.3 μM, but its selectivity was rather limited [60].

## 4. Alkaloids

**Figure 18:**
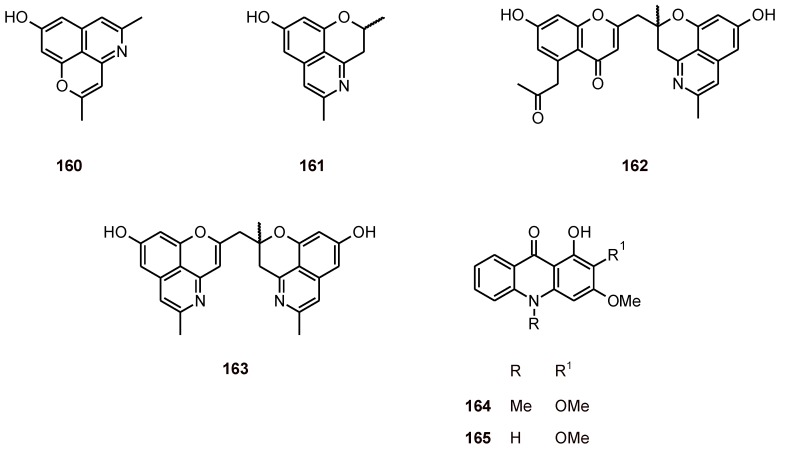
Structures of alkaloids **160**-**165**.

Cassiarin A (**160**) (Figure 18) from the leaves of *Cassia siamea* (Leguminosae) showed promising antimalarial activities. Cassiarin A had inhibitory effects against *P. falciparum* (IC_50_ = 0.02 μM). Antimalarial activity was assessed *in vivo* using the 4-day suppressive test procedure. The ED_50_ value of cassiarin A was 0.17 [61]. In contrast, cassiarins C-E (**161**-**163**) exhibited moderate antiplasmodial activity (IC_50_ values of 24.2 to 2.3 µM) and no cytotoxicity (IC_50_> 100 µM) [62].

Acridone alkaloids have been isolated from the fruits of *Zanthoxylum leprieurii* (Rutaceae), and among them arborinine (**164**) and xanthoxoline (**165**) (Figure 18) exhibited a good *in vitro* antiplasmodial activity against the *P. falciparum* strain 3D7, with IC50 values of 15.8 and 17.0 μM, respectively [63].

**Figure 19:**
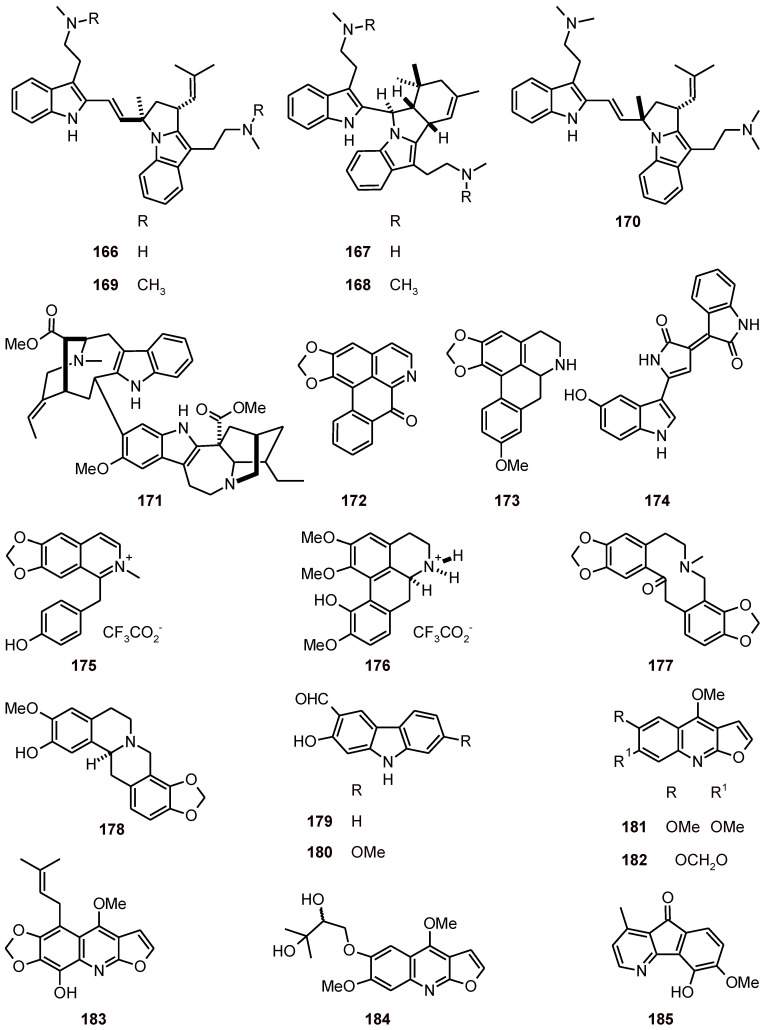
Structures of alkaloids **166**-**185**.

Flinderole A (**166**) and isoborreverine (**167**) have been isolated from *Flindersia acuminata* (Rutaceae) and dimethylisoborreverine (**168**), flinderoles B (**169**), and C (**170**) (Figure 19) have been isolated from *Flindersia ambionensis*. These indole alkaloids were found to have selective antiplasmodial activities, with IC_50_ values of 0.08 to 1.42 μM against the *P. falciparum* strain Dd2 and with selectivity assessed using the mammalian cell line HEK-293 [64]. In addition, the antiplasmodial activities of alkaloids **166-170** and voacamine (**171**) were evaluated using *P. falciparum* strains with different drug-resistance profiles (3D7, FCR3, HB3, and K1). Some differences in the IC_50_ values between the strains were observed. Among these alkaloids, including lirodenine (**172**) and xylopine (**173**), the dimethylisoborreverine (**168**) was the most active, with IC_50_ values between 0.02 μM and 0.81 μM [65].

Violacein (**174**) is a violet pigment extracted from the gram-negative bacterium *Chromobacterium violaceum*. It presents bactericidal, tumoricidal, trypanocidal, and antileishmanial activities. In addition, at micromolar concentrations it efficiently killed chloroquine-sensitive (3D7) and -resistant (S20) *P. falciparum* strains *in vitro*, inhibited parasitemia *in vivo*, even after parasite-establishment; and protected *P. chabaudi chabaudi*-infected mice from a lethal challenge [66]. 

A benzylisoquinoline alkaloid **175** and an aporphine alkaloid **176** (Figure 19) isolated from *Doryphora sassafras* (Monimiaceae), when tested against these same *P. falciparum* strains, exhibited IC_50_ values of 3.0 and 4.4 μM, respectively. Compound **175 **was tested for cytotoxicity toward a human embryonic kidney cell line (HEK293) and showed no activity at 120 μM [67]. The alkaloidal components of the Bhutanese medicinal plant *Corydalis calliantha* (Fumariaceae), which is used for the treatment of malaria, have been assessed. Among them, protopine (**177**) and the cheilanthifoline (**178**) showed promising *in vitro* antiplasmodial activities against *P. falciparum*, both wild type (TM4) and multidrug-resistant (K1) strains, with IC_50_ values of 2.78 to 4.29 µM [68]. Alkaloids **179** and **180** have been isolated from *Clausena harmandiana* (Rutaceae) and they showed antiplasmodial activity with IC_50_ values of 15.5 and 12.2 µM, respectively, against the *P. falciparum* strain K1 [69]. Furoquinoline alkaloids (**181-184**) have been obtained from Teclea afzelii (Rutaceae). When evaluated against P. falciparum (NF54) in vitro, 3 µM of compounds **181-184** showed a partial suppression of parasitic growth [70]. The azafluorenone 5-hydroxy-6-methoxyonychine (**185**) obtained from *Mitrephora diversifolia* (Annonaceae) was shown to be active against *P. falciparum* strains 3D7 and Dd2, with IC_50_ values of 9.9 and 11.4 μM, respectively [71].

**Figure 20:**
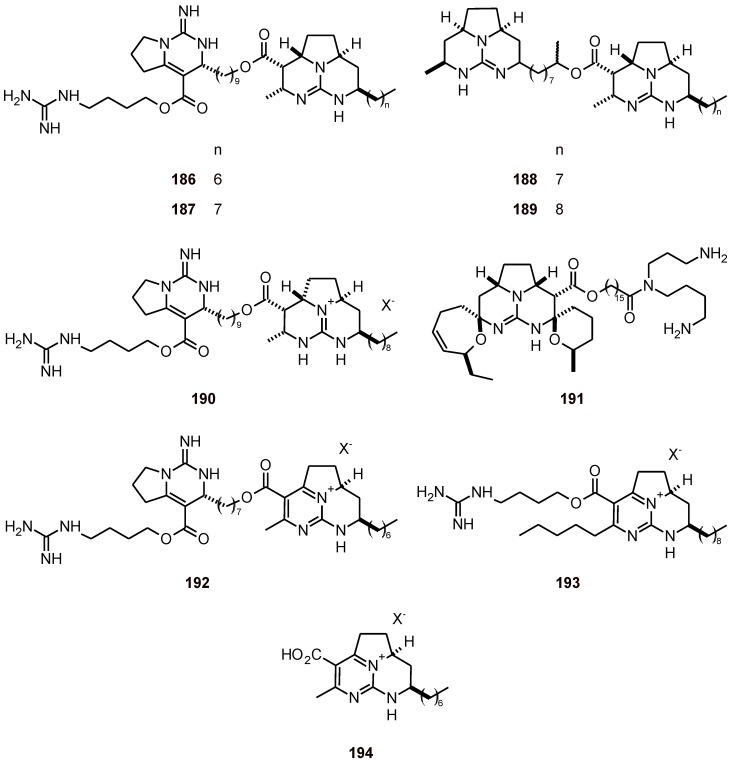
Structures of guanidine alkaloids **186-194.**

**Figure 21:**
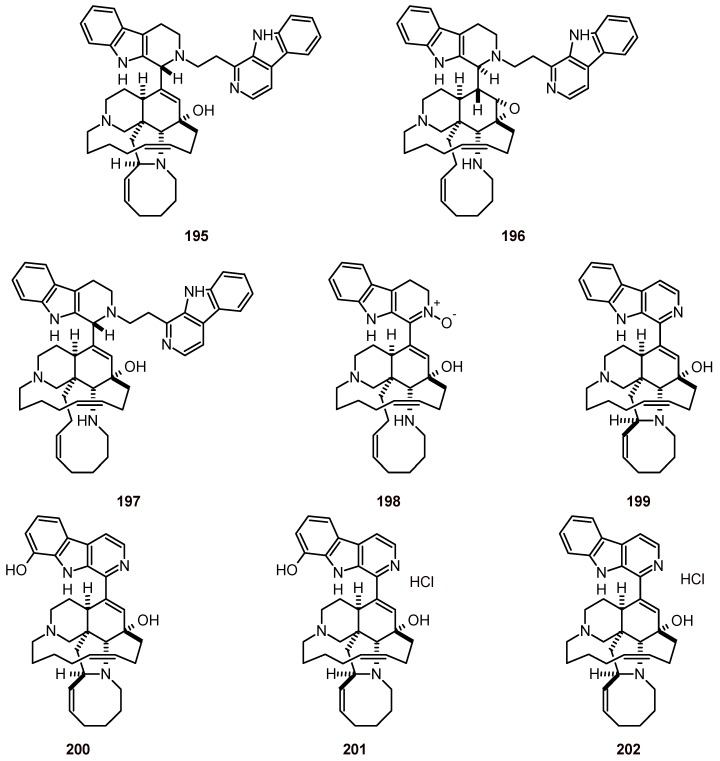
Structures of alkaloids **195-202.**

Nine guanidine alkaloids **186**-**194** (Figure 20) from Caribbean marine sponges, including *Monanchora arbuscula* and *Clathria calla*, were evaluated to determine their activities against human cancer cell lines and malaria protozoa; they exhibited IC_50_ values of 0.1 to 4.5 μM using the *P. flaciparum* strain FcB1 [72]. Zamamidines **195**-**198** and manzamine A (**199**) (Figure 21) have been isolated from the marine sponge species *Amphimedon* and have been shown to exhibit inhibitory activities against *T. brucei* (IC_50_: 1.4, 1.4, 0.4, 7.9, and 0.1 µM, respectively), and *P. falciparum* (IC_50_: 9.6, 16.3, 0.8, 12.4, and 1.8 µM, respectively) *in vitro* [73]. 

*Acanthostrongylophora ingens* has yielded (+)-8-hydroxymanzamine A (**200**), (+)-manzamine A (**199**), (+)-8-hydroxymanzamine A hydrochloride (**201**), and (+)-manzamine A hydrochloride (**202**). Compounds **200** and **201** showed equally potent *in vitro* antiplasmodial activity against chloroquine-sensitive (D6) and -resistant (W2) strains of *P. falciparum* (IC_50_ = 34.6 and 36.6 nM vs. 47.8 and 60.7 nM, respectively), while **199** was >3-fold less potent than **202** (IC_50_ = 37.9 and 47.1 nM vs. 10.5 and 12.5 nM, respectively) [74]. 

**Figure 22:**
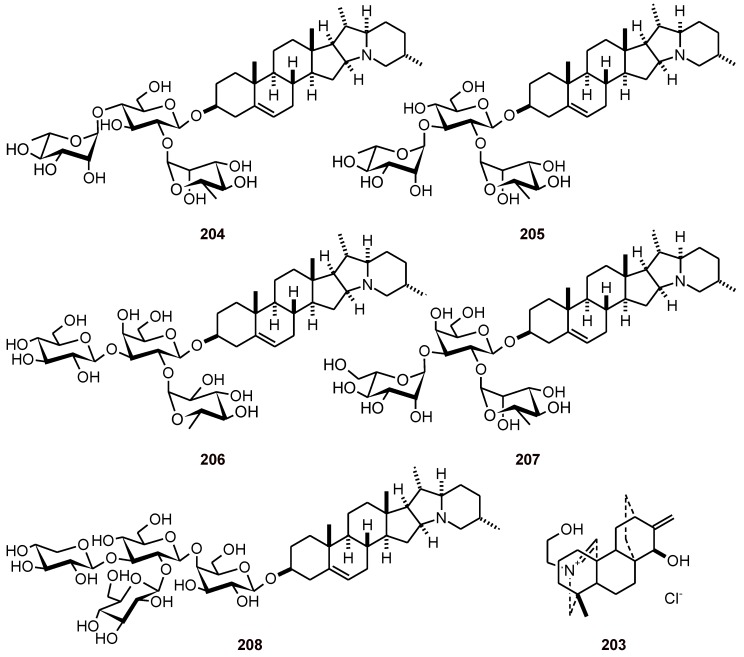
Structures of alkaloids **203**-**208**.

Atisinium chloride (**203**) (Figure 22) is the major alkaloid from *Aconitum orochryseum* (Ranunculaceae). It was tested for *in vitro* antiplasmodial activity against the malarial *P. falciparum* strains TM4/8.2 (wild type) and K1CB1 (chloroquine- and antifolate-resistant), and was shown to have good antiplasmodial activities, with IC_50_ values of 4.0 and 3.6 µM, respectively, against the TM4 strain and the K1 strain of *P. falciparum* [75].

Glycoalkaloids have been isolated from Solanaceae species, and five of them, chaconine (**204**), solanine (**205**), solamargine (**206**), solasonine (**207**), and tomatine (**208**) (Figure 22), were evaluated against *P. yoelii* 17XL in mice at different concentrations with a 4-day parasitemia suppression test. Chaconine (**204**) showed a dose-dependent suppression of malaria infection, with an ED_50_ of 4.49 mg/kg; therapeutic index (TI) **~**9. At a dose of 7.50 mg/kg, the percent parasitemia suppression values of chaconine, tomatine, solamargine, solasonine, and solanine were 71.38, 65.25, 64.89, 57.47, and 41.30%, respectively. At 3.75 mg/kg, the percent parasitemia suppression of chaconine was 42.66% [76]. 

**Figure 23:**
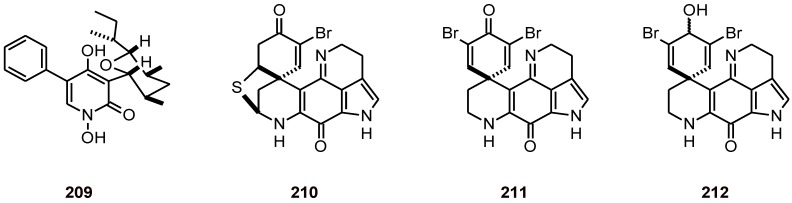
Structures of alkaloids **209**-**212**.

The pyridinone alkaloid **209** (Figure 23) has been isolated from an EtOAc extract of a culture medium of the fungus *Septoria pistaciarum.* It exhibited excellent *in vitro* antiplasmodial activity against chloroquine-sensitive (D6) and -resistant (W2) strains of *P. falciparum* (IC_50_ values of 0.9 and 0.5 µM, respectively) and cytotoxic activity toward Vero cells [77]. 

Pyrroloiminoquinone alkaloids, discorhabdins A (**210**) and C (**211**), and dihydrodiscorhabdin C (**212**) (Figure 23), have been isolated from a deep-water Alaskan sponge species of the genus *Latrunculia* (Latrunculiidae)*.* These alkaloids exhibited anti-HCV activity, antiplasmodial activity against *P. falciparum* strains D6 and W2 (IC_50_ values of 53, 2800, and 170 nM vs. 53, 2000, and 130 nM, respectively), and selective antimicrobial activity. Although compounds **210 **and **212 **displayed potent and selective *in vitro* antiprotozoal activity, *P. berghei*-infected mice did not respond to these metabolites due to their toxicity *in vi*v*o* [78]*.*

**Figure 24:**
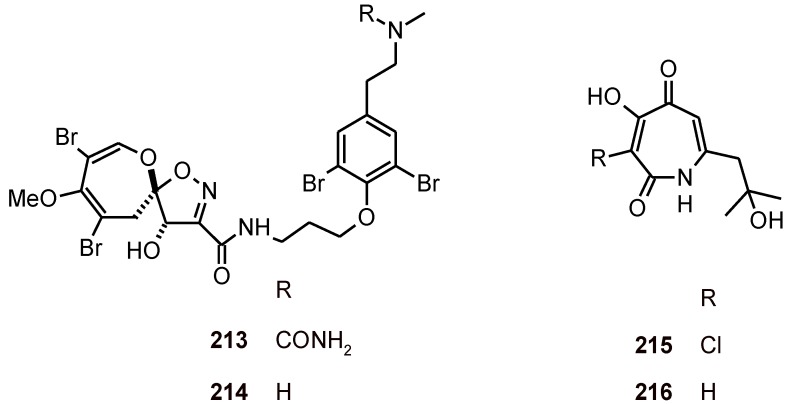
Structures of alkaloids **213**-**216**.

The bromotyrosine alkaloids psammaplysin G (**213**) and psammaplysin F (**214**) (Figure 24) have been isolated from the marine sponge *Hyattella* sp. (Spongiidae). When tested against two different strains of the parasite *P. falciparum* (Dd2 and 3D7), **214 **displayed IC_50_ values of 1.4 and 0.87 μM, respectively, while **213 **showed 98% inhibition at 40 μM against a chloroquine-resistant (Dd2) strain of *P. falciparum* [79]. 

Fermentation culture from the endophytic fungus *Pestalotiopsis* sp. yielded pestalactams A (**215**) and B (**216**) (Figure 24), which were tested against two different strains of the malaria parasite *P. falciparum* (3D7 and Dd2) and parasite growth inhibition of ~16-41% was achieved at 25 µM. Citotoxicity toward mammalian cell lines (MCF-7 and NFF) was also evaluated, and modest *in vitro* activity in all assays was observed [80].

**Figure 25:**
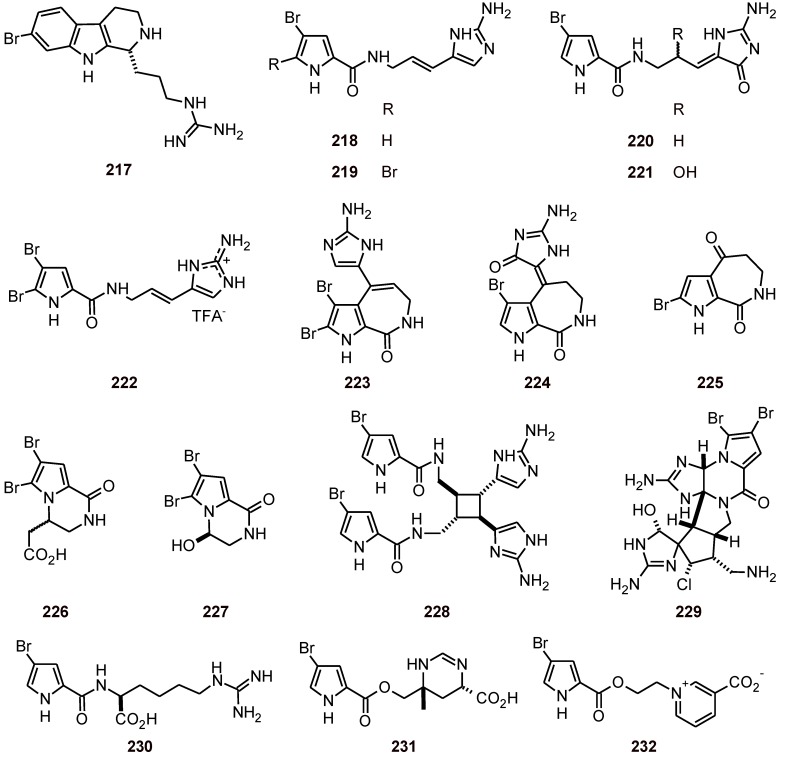
Structures of β-carboline and bromopyrrole alkaloids **217**-**232**.

The β-carboline (+)-7-bromotrypargine (**217**) (Figure 25) has been isolated from the marine sponge *Ancorina* sp. (Ancorinidae). This alkaloid was tested against both a chloroquine*-*resistant (Dd2) and chloroquine-sensitive (3D7) *P. falciparum* strains. Preliminary toxicity toward human cells was investigated using a human embryonic kidney cell line (HEK293), and **217** had IC_50_ values of 5.4 μM (Dd2) and 3.5 μM (3D7), and was not cytotoxic toward the HEK293 cell line at up to 80 μM [81].

Bromopyrrole alkaloids **218**, **220**, **223**, **224**, **226**, **228**, **229**, and **232** (Figure 25) obtained from marine sponges in the genera *Axinella* (Axinellidae) and *Agelas* (Agelasidae) have been screened *in vitro* against four parasitic protozoa, *i.e.*, two *Trypanosoma* species (*T. brucei rhodesiense* and *T. cruzi*), *Leishmania donovani* and *P. falciparum* (strain K1). Longamide B (**226**) and dibromopalau’amine (**229**) have been shown to be promising trypanocidal and antileishmanial agents, while dispacamide B (**220**) and spongiacidin B (**224**) showed the highest antiplasmodial activity (IC_50_ values of 3.3 and 3.4 µM, respectively). In addition, an evaluation of the activity of the alkaloids (**218-232**) tested against three different enzymes (*Pf*FabI, *Pf*FabG, *Pf*FabZ), that are involved in *de novo* fatty acid biosynthesis in *P. falciparum* (*Pf*FAS-II), identified bromopyrrolohomoarginin (**230**) as a potent inhibitor of *Pf*FabZ. Tests against the mammalian L6 cells revealed important clues regarding the therapeutic index of the metabolites [82].

## 5. Peptides and Macrocyclic Compounds

**Figure 26:**
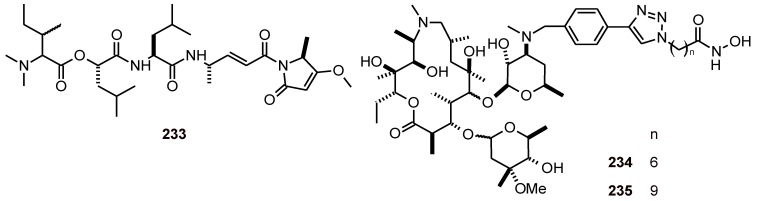
Structures of peptide and macrocyclic compounds **233**-**235**.

Gallinamide A (**233**) (Figure 26) from the cyanobacteria *Schizothrix sp*. (Schizotrichaceae) showed a reasonably effective antimalarial potency against the *P. falciparum* strain W2 (IC_50_ 8.4 *μ*M) and cytotoxicity toward mammalian Vero cells (IC_50 _10.4 *μ*M). However, it did not show *in vitro* cytotoxicity toward NCI-H460 human lung tumor or neuro-2a mouse neuroblastoma cell lines at the highest concentration tested (16.9 *μ*M) [83].

The macrocycles **234 **and **235** (Figure 26) are histone deacetylase inhibitors (HDACi) that cause a diverse range of responses in biological systems [84]. The antiparasitic capabilities of these macrocyclic HDACi were determined against malarial and leishmanial pathogens. Antiparasitic activities of macrocyclic HDACi derived from macrolide skeletons are dependent on the length (*n*) of the spacer group that separates their zinc-binding and surface-recognition moieties. Antimalarial activities peak when *n*=6 (IC_50_ **234**: 95 nM), whereas antileishmanial activities are optimum when *n*=8–9 (IC_50_ **235**: ~3.3 µM). This observation could facilitate the identification of other HDACi that are more selective for either parasite [84].

## 6. Phenylalkanoids

### 6.1. Phenylpropanoids

**Figure 27:**
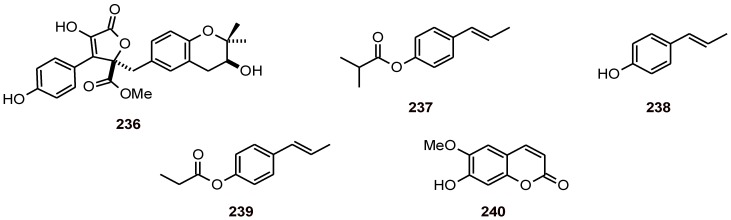
Structures of phenylpropanoids **236**-**240**.

The lignan butyrolactone **236** (Figure 27) from the fungus *Aspergillus terreus* BCC 4651 showed antiplasmodial activity against the *P. falciparum* strain K1 with an IC_50_ value of 18 µM [85]. The main constituent (39.0%) of the volatile constituents of *Daucus crinitus* was isochavicol isobutyrate (**237**). This compound, together with isochavicol (**238**) and isochavicol propionate (**239**), exhibited antiplasmodial activity against *P. falciparum* (strain FcB1) with IC_50_ values of 68.7, 14.2 and 70.0 µM, respectively [86]. In contrast, coumarin **240** did not exhibit any significant antiplasmodial activity (IC_50_ value of 146.9 µM against strain D10) [38].

### 6.2. Phenylethanoids

**Figure 28:**
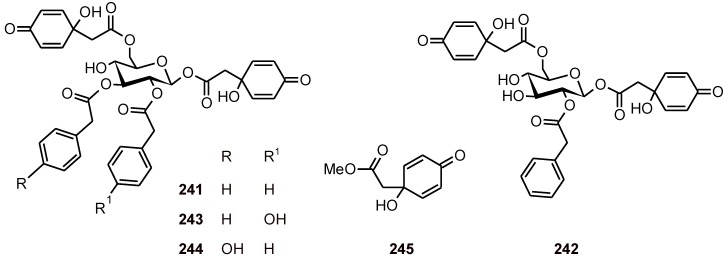
Structures of phenylethanoids **241**-**245**.

*Jacaranda glabra* (Bignoniaceae) yielded phenylethanoid glucosides (**241**-**244**) and jacaranone (**245**) (Figure 28), which were found to be active *in vitro* against the *P. falciparum* strain K1 (IC_50 _**241**: 1.5; **242**: 1.0; **243**: 0.8; **244**: 0.8 and **245**: 7.3 μM). All of the compounds except for **241** showed low cytotoxicity toward L-6 cells [87].

### 6.3. Phenylmethanoids, benzylesters, and phenolics

**Figure 29:**
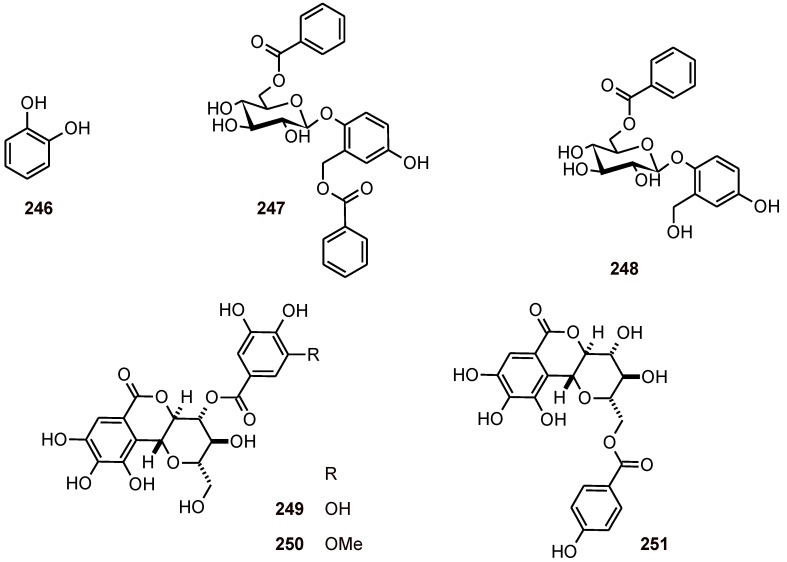
Structures of phenylmethanoids and phenolics **246**-**251**.

A phenol **246** and two phenolic glycosides **247** and **248** (Figure 29) have been isolated from *Flacourtia indica* (Flacourtiaceae), and they have been shown to have antiplasmodial activity toward the W2 strain of *P. falciparum* (IC_50_ values of 0.7 to 27 µM) [88]. Norbergenin derivatives **249-251** (Figure 29) isolated from the stem bark of *Diospyros sanza-minika* (Ebenaceae) were evaluated for their *in vitro* activity against the *P. falciparum* K1 and cytotoxicity toward MRC-5 cells (**249**: IC_50_ 8.4 μM; CC_50_> 137.4 μM, **251**: IC_50_ 11.3 μM; CC_50_> 147.5 μM, **250**: IC_50_ 1.3 μM; CC_50_ 51.5 μM). Compound **251 **possesses an *O*-*p*-hydroxybenzoyl group at C-11, while **249** and**250** have *O*-galloyl, and *O*-(3′-methylgalloyl) groups, respectively, at C-4, which may play an important role in their antimalarial activity [89].

## 7. 4-Aryl-3,4-dihydrocoumarins

**Figure 30:**
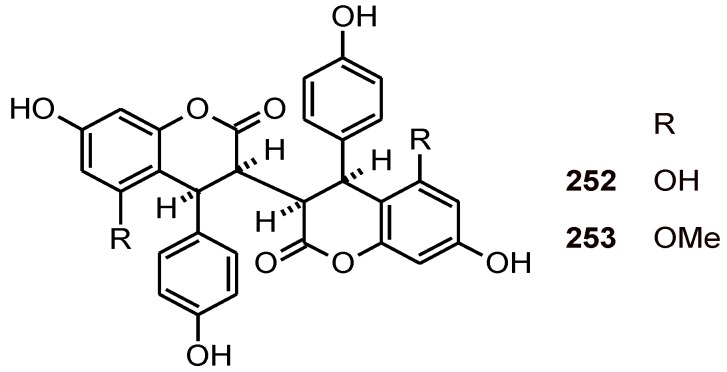
Structures of 4-aryl-3,4-dihydrocoumarin dimers **252** and **253**.

In addition to antiplasmodial biflavonoids, *Ormocarpum kirkii* (Papilionaceae) yielded 4-aryl-3,4-dihydrocoumarins **252** and **253**, which were active against the *P. falciparum* strain K1 (IC_50_ values of 39.5 and 21.1 μM, respectively) (Figure 30) [90].

## 8. Xanthones, Naphthopyrones, and Analogues Vismiones

**Figure 31:**
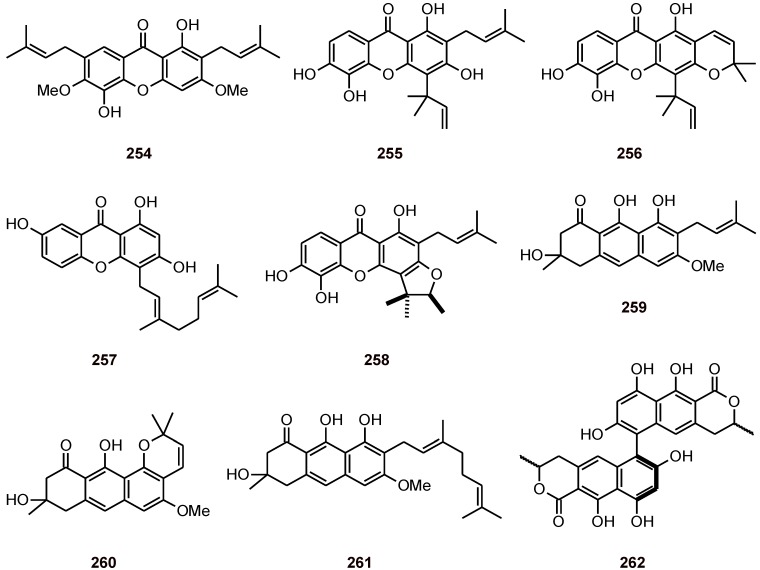
Structures of xanthones, naphthopyrones, and analogues vismiones **254**-**262**.

The xanthone 1,5-dihydroxy-3,6-dimethoxy-2,7-diprenylxanthone (**254**) (Figure 31) showed selective activity against *P. falciparum* with an IC_50_ value of 7.25 µM. When screened for activity against *T. cruzi*, *T. brucei*, *L. infantum* (Ghana strain), *S. aureus*, and *E. coli*, and for cytotoxicity against MRC-5 cells, it showed IC_50_ values >64 µM [50]. Xanthones **255**-**258** and their analogues **259**-**261** have been isolated from *Cratoxylum maingayi* and *Cratoxylum cochinchinense* (Clusiaceae) [91]. These compounds showed antiplasmodial activity against *P. falciparum* at concentrations of 11.0 to 1.9 µM. Most of these compounds also showed cytotoxicity toward the NC1-H187 cancer cell line [91].

Aschernaphthopyrone A (**262**) has been isolated from the scale insect pathogenic fungus *Aschersonia paraphysata*, and its antiplasmodial activity against the *P. falciparum* strain K1 (IC_50_ = 7.3 μM) is higher than that of hopene triterpene **143** (Figure 15) isolated from the same fungus [51].

## 9. Anthraquinones and Anthrones

**Figure 32:**
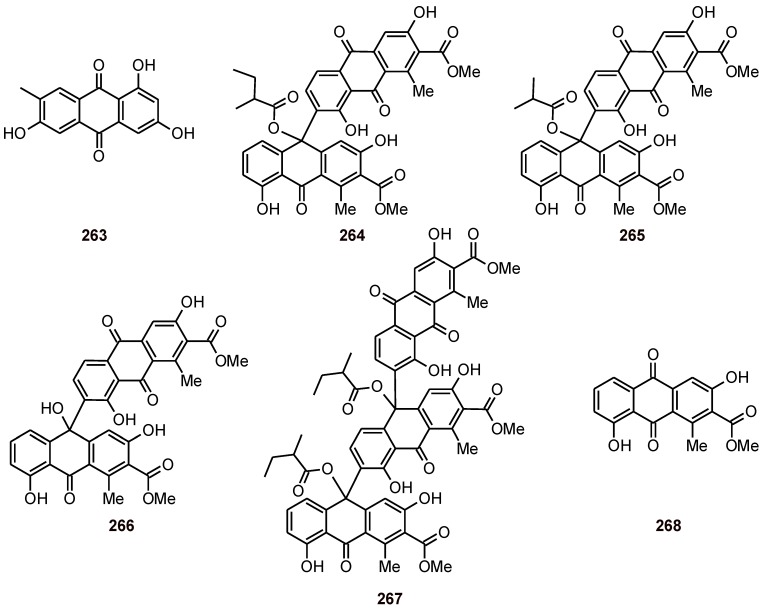
Structures of anthraquinones and anthrones **263-268**.

Demethylmacrosporine (**263**) (Figure 32) isolated from *Rumex obtusifolius* (Polygonaceae) showed significant activity in the FBIT (Ferriprotoporphyrine biocrystallization inhibition test, IC_50_ = 0.3 μM) [21]. The anthrone–anthraquinones scutianthraquinones A, B, and C (**264**–**266**), and the bisanthrone–anthraquinone scutianthraquinone D (**267**) have been isolated from the bark of *Scutia myrtina* (Rhamnaceae). Compounds **264**-**267** and **268** (aloesaponarin I, which was isolated from *Aloe saponaria* - Asphodelaceae) exhibited good antiplasmodial activities against the *P. falciparum* Dd2 (IC_50_ values of 1.1 to 5.6 μM), while compounds **264**, **265**, and **267** also exhibited good antiplasmodial activity against the *P. falciparum* strain FCM29 (IC_50_ values of 1.2 to 5.6 μM), these compounds also showed weak antiproliferative activity against the ovarian cancer cell line A2780 [92].

## 10. Halenaquinone Derivatives

**Figure 33:**
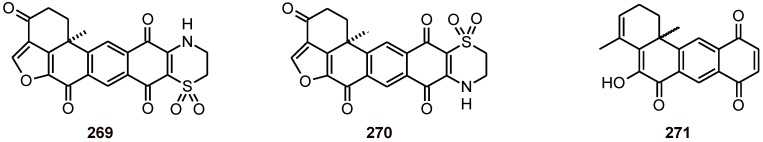
Structures of halenaquinone derivatives and analogues **269**-**271**.

Compounds **269**-**271** (Figure 33) were the most active of a series of halenaquinone derivatives from South Pacific marine sponges of the genus *Xestospongia* (Petrosiidae). The exhibited antiplasmodial activity did not depend on the chloroquine-sensitivity of the strain tested, since there was no significant difference between the IC_50_ values for strains FcB1 (IC_50_ 1.1, 3. 9, and 9.2 μM, respectively) and 3D7 (IC_50_ 1. 7, 4.1, and 10.9 μM, respectively). The three active compounds were also active in protein farnesyltransferase bioassays, which may provide insight into their mode of action against *P*. *falciparum* [22].

## 11. Endoperoxides, Peroxides and Other Polyketides from Sponges

**Figure 34:**
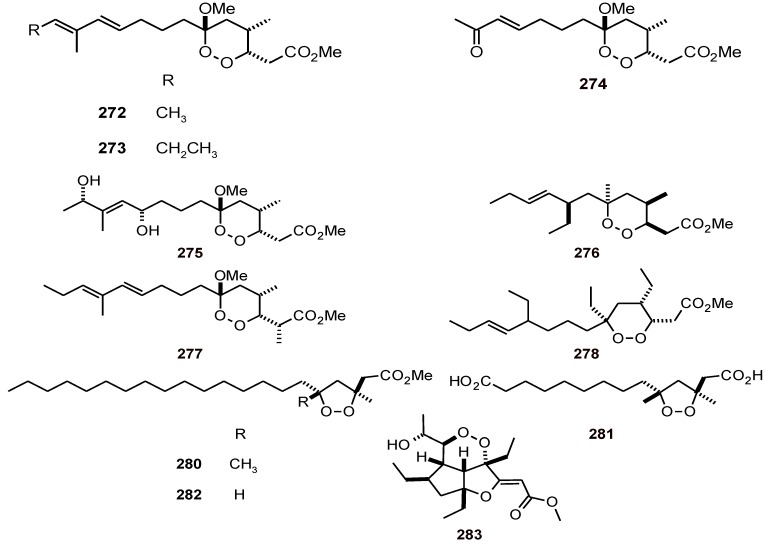
Structures of peroxide polyketides **272-278 **and **280-282**.

**Figure 35:**
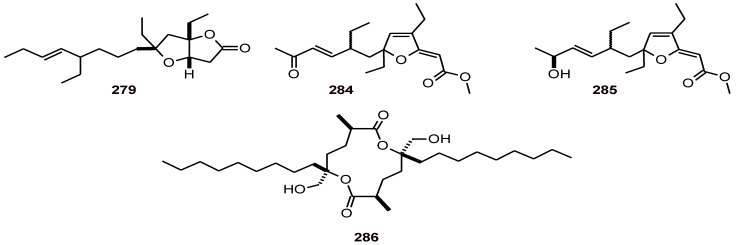
Structures of other polyketides **279** and **284**-**286 **from sponges.

Endoperoxyketal polyketides manadoperoxides A-D (**272**-**275**) (Figure 34) have been isolated from the Indonesian sponge *Plakortis* cfr*. Simplex* (Plakinidae), and were assayed *in vitr*o against the D10 and W2 strains of *P. falciparum*, where they showed good antimalarial activity (IC_50_ values of 4.5 to 10.4 and 2.3 to 7.9 µM, respectively) compared to those of plakortin (**276**, IC_50_ value of 0.9 and 0.4 µM, respectively) and peroxyplakoric B3 ester (**277**, *P. falciparum* strain FCR3, IC_50_ = 1.1 µM), the latter of which differs from manadoperoxide B (*P. falciparum* strain FCR3, IC_50_ = 6.8 µM) by only minor structural details. This difference in the antiplasmodial activity has been explained on the basis of a model for the interaction of 1,2-dioxanes with heme and the production of C-centered radicals that are toxic to the parasite. For the manadoperoxides, either the endoperoxide linkage is inaccessible to the heme iron or the O1 radical cannot evolve to produce a C-centered radical [93]. Another polyketide-peroxy, plakortide F (methyl ester **278**), has been isolated from a species of *Plakortis* (Plakinidae) from Jamaica. It exhibited good *in vitro* antiplasmodial activity (IC_50_ values of 3.4 and 2.5 µM against *P. falciparum* D6 and W2 strains, respectively), whereas a non peroxide-polyketide, plakortone D (**279**) (Figure 35), exhibited IC_50_ values of 7.8 and 8.7 µM, respectively [94]. Five-membered-ring polyketide endoperoxides **280** and **281**, and a cyclic peroxide **282** (Figure 34) have been isolated from the sponge *Plakortis halichondrioides* (Plakinidae). Biological screening of these cycloperoxides for cytotoxic activity against various human tumor cell lines revealed that compounds **281** and **282** are very active. In assays for antiplasmodial activity, compounds **280**-**282** also showed good activity against the pathogenic microbe *P. falciparum* (IC_50_ values of 4.0, 0.3 and 3.0 µM, respectively). Compound **280** also showed antitubercular activity against *Mycobacterium tuberculosis* (IC_50_ values of 62 and 71 µM, respectively) [95].

Gracilioethers A-C (**283**-**285**) (Figure 34 and Figure 35) have been isolated from the marine sponge, *Agelas gracilis* (Agelasidae), and they showed antimalarial activity against the ItG strain of *P. falciparum* with IC_50_ values of 1.6 to 31 μM, and gracilioether B (**284**) also showed antileishmanial activity [96]. Malyngolide dimer (**286**) (Figure 35) has been isolated from the marine cyanobacterium *Lyngbya majuscule* (Oscillatoriaceae). It exhibited moderate *in* v*itro* antiplasmodial activity against chloroquine-resistant *P. falciparum* (W2, IC_50_ = 19 μM), but roughly equivalent toxicity toward H-460 human lung cell lines [97].

## 12. Acetylenes

**Figure 36:**
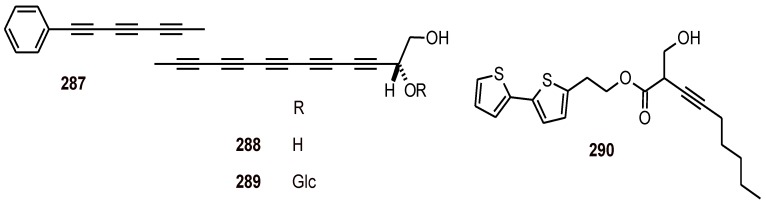
Structures of polyacetylenes **287-290**.

Three acetylenic compounds, 1-phenyl-hepta-1,3,5-triyne (**287**), (*R*)-1,2-dihydroxytrideca-3,5,7,9,11-pentayne (**288**), and its glycoside, 2-β-D-glycopyrasyloxy-1-hydroxytrideca-3,5,7,9,11-pentayne (**289**) (Figure 36) have been isolated from *Bidens pilosa* (Compositae)*.* Among other activities, these compounds showed potential antimalarial activity *in vitro* (determined spectrophotometrically by measuring the activity of the pLDH, in control and drug-treated cultures or using the Nagel method against the RCR-3 strain of *P. falciparum*) [98,99]. Compound **287** when tested *in vitro* showed an IC_50_ = 37.2 µM (*P. falciparum* strain NF54), while compound **288** showed an IC_50_ = 1.8 µM. In *in vivo* assays the average 32.8% malaria parasite growth (*P. berhei* strain NK-65) diminished to 12.1% by administration of a dose 0.8 mg/kg of **288** for four days in mice [98,99].

The roots of *Tagetes erecta* (Compositae) yielded 2-hydroxymethyl-non-3-ynoic acid 2-[2,2']-bithiophenyl-5-ethyl ester (**290**). This compound was evaluated for its *in vitro* antiplasmodial activity using the schizont maturation inhibition assay, and showed significant schizonticidal activity against both chloroquine-sensitive (MRC-pf-2) and -resistant (MRC-pf-56) strains of *P. falciparum* with IC_50_ values of 26 and 53 nM, respectively [100]. 

## 13. β-Resorcylic Acid Lactones

**Figure 37:**
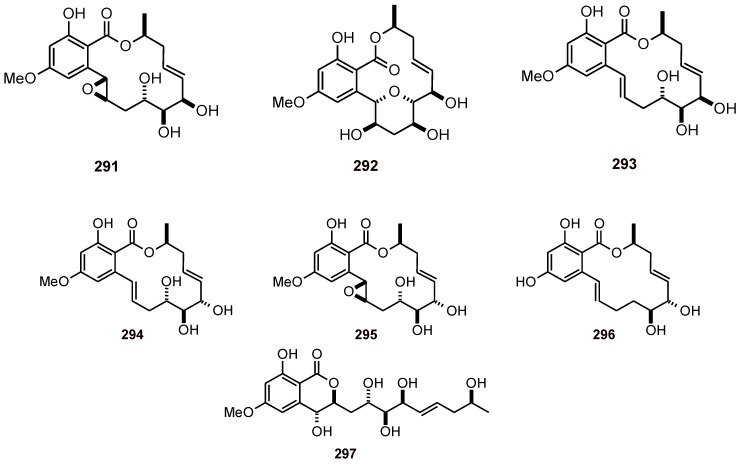
Structures of *β*-resorcylic acid lactones **291**-**297**.

The *β*-resorcylic acid lactones paecilomycins (**291**-**294**), aigialomycin B (**295**), aigialomycin D (**296**), and aigialomycin F (**297**) (Figure 37) were isolated from the mycelial solid culture of *Paecilomyces* sp. (Trichocomaceae, fungus). These lactones exhibited antiplasmodial activity against the 3D7 line of *P. falciparum*, and **294 **and **297** were the most active, with IC_50_ values of 20.0 and 10.9 nM, respectively, whereas compounds **293-295 **showed good activity against the *P. falciparum* strain Dd2, with IC_50_ values of 1.7 to 13.5 µM [101].

## 14. Depsidones

**Figure 38:**
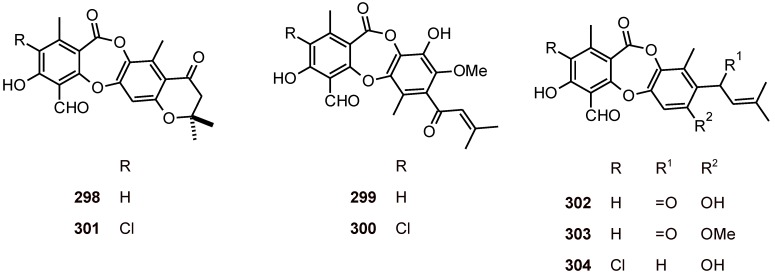
Structures of depsidones **298-304**.

The depsidones mollicellins B (**298**), C (**299**), E (**300**), K-M (**301**-**303**), and J (**304**) (Figure 38) isolated from *Chaetomium brasiliense* (Chaetomiaceae) exhibited *in vitro* antimalarial activity against *P. falciparum (*K1 multidrug-resistant, IC_50_ = 12.3, 22.0, 7.2, 7.0, 3.1, 8.6, and 12.2 µM, respectively). These compounds also showed *in vitro* cytotoxicity against KB, BC1, and NCI-H187 cells, as well as five cholangiocarcinoma cell lines [102].

## 15. Benzophenones

**Figure 39:**
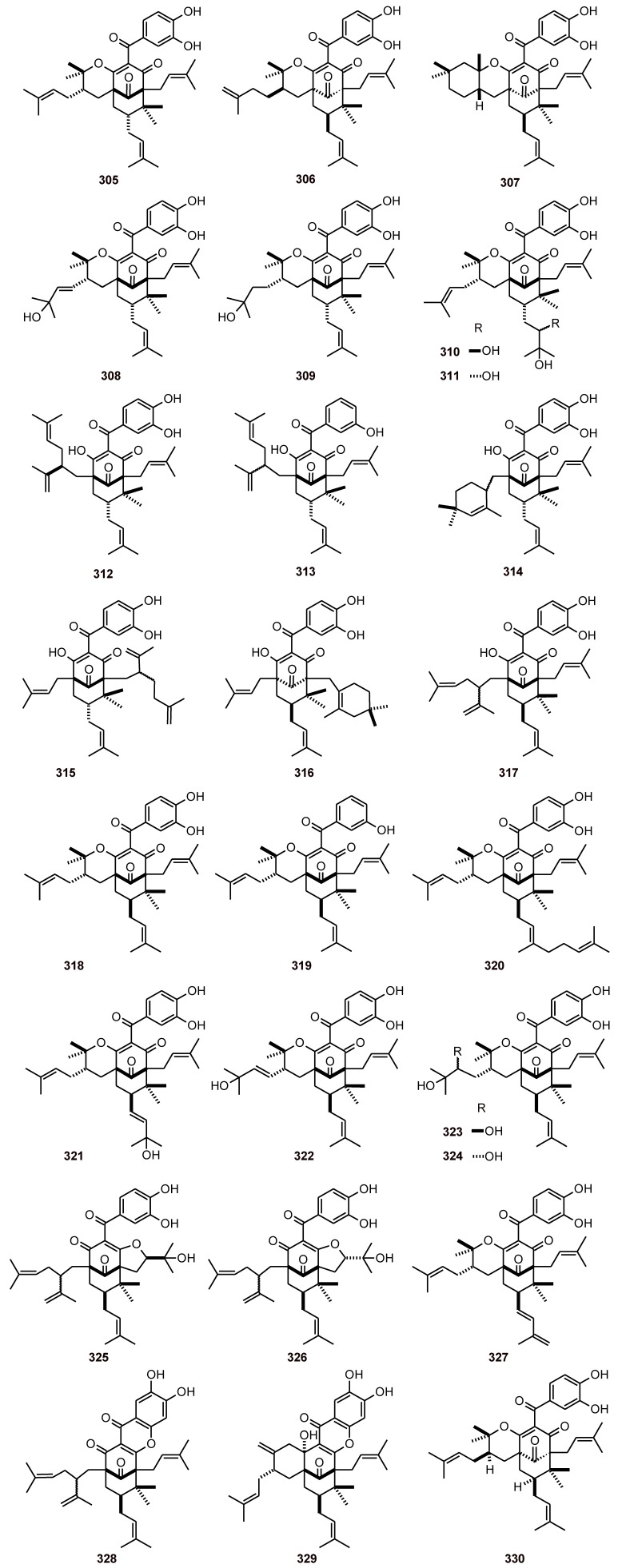
Structures of benzophenones **305**-**330**.

A series of antiplasmodial benzophenones **305**-**318** (Figure 39), with IC_50_ values of 3.3 to 37.2 μM, were isolated from *Moronobea coccinea* (Clusiaceae)*.* The benzophenone cytotoxicities were also evaluated toward the human cell line MRC-5 [103]. Isoxanthochymol (**330**) from *Garcinia* spp. (Clusiaceae) exhibited broad but non-selective antiprotozoal activity, with an IC_50_ value of 4.47 µM against *P. falciparum*, and was also cytotoxic (IC_50_ 7.46 µM) [50]. *Symphonia globulifera* (Clusiaceae) also contains polycyclic polyprenylated acylphloroglucinol compounds **317-327** and two oxidized derivatives **328** and **329** (Figure 39). All compounds showed antiplasmodial activity when evaluated *in vitro* against a chloroquine-resistant strain of *P. falciparum* (FcB1), with IC_50_ values of 2.1 to 10.1 µM [104].

## 16. Miscellaneous Compounds

### 16.1. Diterpene-benzoate macrolides and analogues

**Figure 40:**
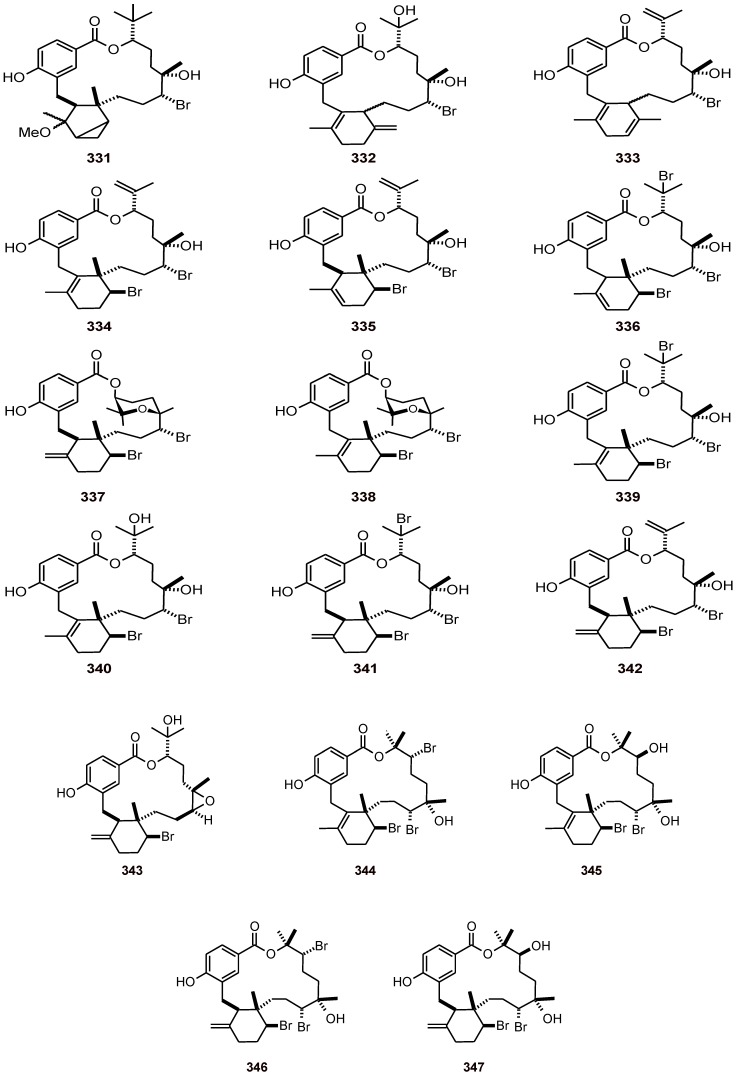
Structures of diterpene-benzoate macrolides and analogues **331**-**347.**

Several bromophycolides **331**-**347** (Figure 40) with a diterpene-benzoate macrolide carbon skeleton have been obtained from the red alga *Callophycus serratus* (Solieriaceae), and have been shown to exhibit excellent to moderate antiplasmodial activity against the *P. falciparum* strain 3D7 (IC_50_ values of 0.3 to 56 μM) [105].

### 16.2. Strobilurins

**Figure 41:**
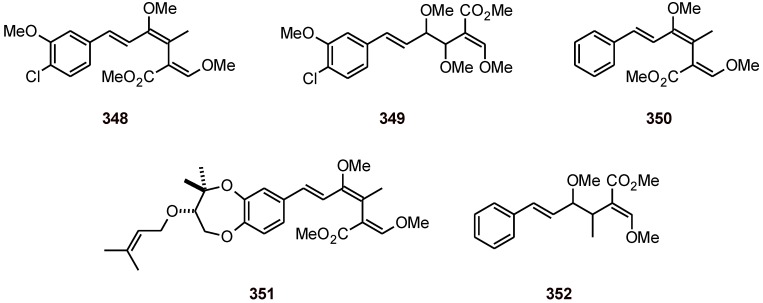
Structures of strobilurins **348**-**352**.

Strobilurins **348**-**352**, two of which (**348** and **349**) are monochlorinated (Figure 41), have been obtained from the fungus *Favolaschia tonkinensis.* In addition to their antifungal and cytotoxic activities, they also exhibited antiplasmodial activity against the *P. falciparum* strain K1, with IC_50_ values of 0.06 to 10.3 µM [106].

### 16.3. Other compounds

**Figure 42:**
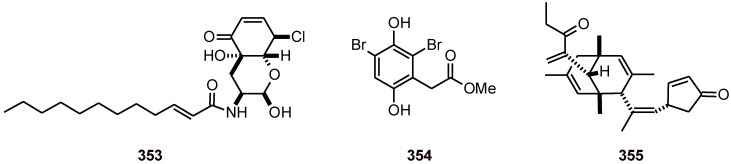
Structures of compounds **353**-**355**.

Isariotin F (**353**) (Figure 42) isolated from the fungus *Isaria tenuipes* also exhibited activity against the malaria parasite *P. falciparum* K1 with an IC_50_ value of 5.1 *μ*M, as well as cytotoxic activities toward cancer cell lines (IC_50_ values of 15.8, 2.4, and 1.6 *μ*M, in KB, BC, and NCI-H187, respectively) and nonmalignant (Vero) cells (IC_50_ = 2.9 *μ*M) [107].

**Figure 43:**
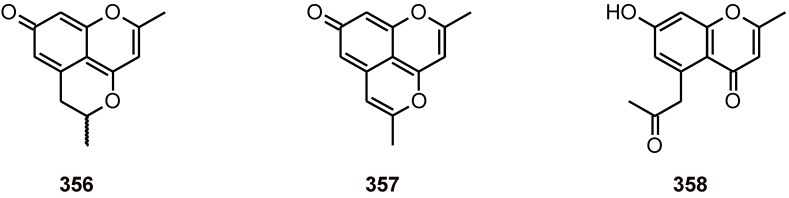
Structures of chromenes **356-358.**

From the marine sponge *Pseudoceratina* sp. (Pseudoceratinidae) was isolated a derivative of homogentisic acid (**354**) (Figure 43), which inhibited a specific protein kinase of *P. falciparum* (Pfnek-1) with an IC50 of around 1.8 μM. This product was active *in vitro* assays against the *P. falciparum* strain FcB1 (IC50 = 12 μM) [108]. 

Compound **355** (Figure 42) has been obtained from the fungus *Emericella rugulosa*. It exhibited good antiplasmodial activity against the *P. falciparum* strain K1 (IC_50_ value of 5.1 μM), antimycobacterial (MIC value of 33.1 μM) activity, and cytotoxicity against three cancer cell lines (IC_50_ value of 3.5, 6.9, and 3.5 μM, in BC1, KB, and NCI-H187, respectively) [109].

Chromenes from *Cassia siamea* (Leguminosae) **356-358** (Figure 43) exhibited good antiplasmodial activity against the *P. falciparum* strain 3D7 (IC_50_ **356**: 2.3 μM; **357**: 4.7 μM; **358**: 8.6 μM) and no cytotoxicity (IC_50_> 100 µM) [62].

**Figure 44:**
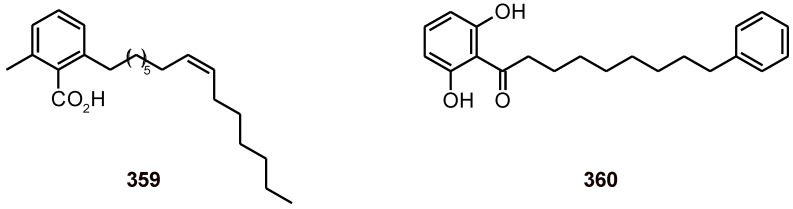
Structures of compounds **359 **and **360**.

The 6-(8'Z-pentadecenyl)-salicylic acid (**359**) (Figure 44) isolated from *Viola websteri* (Violaceae) was found to have antimalarial activity *in vivo*. When tested against *P. berghei* in mice 6-SA, at 5, 10 and 25 mg/kg/day exhibited a significant blood schizonticidal activity, and at these concentrations no marked toxicity was observed in mice [110]. Another polyketide derivative (malabaricone A, **360**) has been isolated from *Knema glauca* (Myristicaceae). It showed moderate cytotoxicity, antituberculosis activity against *M. tuberculosis*, and antiplasmodial activity against the parasite *P. falciparum* strain K1 with an IC_50_ value of 8.6 μM [111].

## 4. Conclusions

A few drugs, alone or in combination—chloroquine, primaquine, mefloquine, halofantrine, artemisinin, atovaquone, among others—have been used in chemotherapy for malaria. However, the evolution of drug- or multidrug-resistance has been a challenge for the effectiveness of such chemotherapy. None of the papers in this review claim to have discovered the next antimalarial drug. Instead, they provide a remarkable diversity of new natural products on which to base the discovery and development of antimalarial drugs. This considerable structural diversity is represented in the 360 relevant structures that we examined (as illustrated in Figures 1-44). Several potent antiplasmodial natural products have been described, and those belonging to alkaloid (manzamine, pyridinone, and pyrroloiminoquinone), polyacetylene, phenylethanoid, anthraquinone, polyketide (endoperoxide), nonpeptide macrocyclic, and β-resorcylic lactone classes have high antiplasmodial activity. Most of the active compounds described here have only been evaluated by *in vitro* assays, few have been evaluated for cytotoxicity, and still fewer have been assayed *in vivo*. The compounds listed (Figures 1-44) have been included based on the potency and/or selectivity of their biological properties, and reflect the tremendous effort that is being devoted to recognizing the potential of natural products as lead compounds in the treatment of malaria.

## References

[1] Watts K.R., Tenney K., Crews P. (2010). The structural diversity and promise of antiparasitic marine invertebrate-derived small molecules. Curr. Opin. Biotechnol..

[2] Singh B., Sung L.K., Matusop A., Radhakrishnan A., Shamsul S.S.G., Cox-Singh J., Thomas A., Conway D.J. (2004). A large focus of naturally acquired *Plasmodium knowlesi* infections in human beings. Lancet.

[3] Vogel G. (2010). Infectious disease - New map illustrates risk from the 'other' malaria. Science.

[4] Fidock D.A. (2010). Drug discovery - Priming the antimalarial pipeline. Nature.

[5] Mendis K., Rietveld A., Warsame M., Bosman A., Greenwood B., Wernsdorfer W.H. (2009). From malaria control to eradication: The WHO perspective. Trop. Med. Intern. Health.

[6] Wells T.N.C., Alonso P.L., Gutteridge W.E. (2009). New medicines to improve control and contribute to the eradication of malaria. Nat. Rev. Drug Discov..

[7] Gamo F.J., Sanz L.M., Vidal J., de Cozar C., Alvarez E., Lavandera J.L., Vanderwall D.E., Green D.V.S., Kumar V., Hasan S., Brown J.R., Peishoff C.E., Cardon L.R., Garcia-Bustos J.F. (2010). Thousands of chemical starting points for antimalarial lead identification. Nature.

[8] Camargo L.M., de Oliveira S., Basano S., Garcia C.R. (2009). Antimalarials and the fight against malaria in Brazil. Ther. Clin. Risk Manag..

[9] Fattorusso E., Taglialatela-Scafati O. (2009). Marine Antimalarials. Mar. Drugs.

[10] Bero J., Frédérich M., Quetin-Leclercq J. (2009). Antimalarial compounds isolated from plants used in traditional medicine. J. Pharm. Pharmacol..

[11] Magadula J.J., Erasto P. (2009). Bioactive natural products derived from the East African flora. Nat. Prod. Rep..

[12] Batista R., Silva A.D.J., de Oliveira A.B. (2009). Plant-derived antimalarial agents: new leads and efficient phytomedicines. Part II. Non-alkaloidal natural products. Molecules.

[13] Sivonen K., Leikoski N., Fewer D.P., Jokela J. (2010). Cyanobactins-ribosomal cyclic peptides produced by cyanobacteria. Appl. Microbiol. Biotechnol..

[14] Mariath I.R., Falcão H.D., Barbosa-Filho J.M., de Sousa L.C.F., Tomaz A.C.A., Batista L.M., Diniz M.F.F.M., Athayde P.F., Tavares J.F., Silva M.S., da Cunha E.V.L. (2009). Plants of the American continent with antimalarial activity. Rev. Bras. Farmacogn..

[15] Gademann K., Kobylinska J. (2009). Antimalarial natural products of marine and freshwater origin. Chem. Rec..

[16] Wright C.W. (2010). Recent developments in research on terrestrial plants used for the treatment of malaria. Nat. Prod. Rep..

[17] Krettli A.U. (2009). Antimalarial drug discovery: screening of Brazilian medicinal plants and purified compounds. Expert Opin. Drug Discov..

[18] Kaur K., Jain M., Kaur T., Jain R. (2009). Antimalarials from nature. Bioorg. Med. Chem..

[19] Ikegami-Kawai M., Arai C., Ogawa Y., Yanoshita R., Ihara M. (2010). Selective accumulation of a novel antimalarial rhodacyanine derivative, SSJ-127, in an organelle of *Plasmodium berghei*. Bioorg. Med. Chem..

[20] Corbett Y., Herrera L., Gonzalez J., Cubilla L., Capson T.L., Coley P.D., Kursar T.A., Romero L.I., Ortega-Barria E. (2004). A novel DNA-based microfluorimetric method to evaluate antimalarial drug activity. Am. J. Trop. Med. Hyg..

[21] Ibáñez-Calero S.L., Jullian V., Sauvain M. (2009). A new anthraquinone isolated from *Rumex obtusifolius*. Rev. Boliv. Quím..

[22] Longeon A., Copp B.R., Roué M., Dubois J., Valentin A., Petek S., Debitus C., Bourguet-Kondracki M.L. (2010). New bioactive halenaquinone derivatives from South Pacific marine sponges of the genus *Xestospongia*. Bioorg. Med. Chem..

[23] Pabón A., Deharo E., Zuluaga L., Maya J.D., Saez J., Blair S. (2009). *Plasmodium falciparum*: Effect of *Solanum nudum* steroids on thiol contents and β-hematin formation in parasitized erythrocytes. Exp. Parasitol..

[24] Krettli A.U., Adebayo J.O., Krettli L.G. (2009). Testing of natural products and synthetic molecules aiming at new antimalarials. Current Drug Targets.

[25] Wein S., Maynadier M., Van Ba C.T., Cerdan R., Peyrottes S., Fraisse L., Vial H. (2010). Reliability of antimalarial sensitivity tests depends on drug mechanisms of action. J. Clin. Microbiol..

[26] Tamura S., Kubata B.K., Syamsurizal, Itagaki S., Horii T., Taba M.K., Murakami N. (2010). New anti-malarial phenylpropanoid conjugated iridoids from *Morinda morindoides*. Bioorg. Med. Chem. Lett..

[27] Soni S., Gupta S. (2009). *In vitro* anti-plasmodial activity of *Enicostemma littorale*. Am. J. Infect. Dis..

[28] Afolayan A.F., Mann M.G.A., Lategan C.A., Smith P.J., Bolton J.J., Beukes D.R. (2009). Antiplasmodial halogenated monoterpenes from the marine red alga *Plocamium cornutum*. Phytochemistry.

[29] Isaka M., Srisanoh U., Veeranondha S., Choowong W., Lumyong S. (2009). Cytotoxic eremophilane sesquiterpenoids from the saprobic fungus *Berkleasmium nigroapicale* BCC 8220. Tetrahedron.

[30] Pedersen M.M., Chukwujekwu J.C., Lategan C.A., van Staden J., Smith P.J., Staerk D. (2009). Antimalarial sesquiterpene lactones from *Distephanus angulifolius*. Phytochemistry.

[31] Efange S.M.N., Brun R., Wittlin S., Connolly J.D., Hoye T.R., McAkam T., Makolo F.L., Mbah J.A., Nelson D.P., Nyongbela K.D., Wirmum C.K. (2009). Okundoperoxide, a bicyclic cyclofarnesylsesquiterpene endoperoxide from *Scleria striatinux* with antiplasmodial activity. J. Nat. Prod..

[32] Kim J.J., Chung I.M., Jung J.C., Kim M.Y., Moon H.I. (2009). *In vivo* antiplasmodial activity of 11(13)-dehydroivaxillin from *Carpesium ceruum*. J. Enzyme Inhib. Med. Chem..

[33] Schmidt T.J., Nour A.M.M., Khalid S.A., Kaiser M., Brun R. (2009). Quantitative structure- antiprotozoal activity relationships of sesquiterpene lactones. Molecules.

[34] Rodríguez I.I., Rodríguez A.D., Zhao H. (2009). Aberrarone: A gorgonian-derived diterpene from *Pseudopterogorgia elisabethae*. J. Org. Chem..

[35] Wei X., Rodríguez A.D., Baran P., Raptis R.G. (2010). Dolabellane-type diterpenoids with antiprotozoan activity from a Southwestern Caribbean gorgonian octocoral of the genus *Eunicea*. J. Nat. Prod..

[36] Sathe M., Ghorpade R., Srivastava A.K., Kaushik M.P. (2010). *In vivo* antimalarial evaluation of gomphostenins. J. Ethnopharmacol..

[37] Sathe M., Kaushik M.P. (2010). Gomphostenins: Two new antimalarial compounds from the leaves of *Gomphostemma niveum*. Bioorg. Med. Chem. Lett..

[38] Mthembu X.S., Van Heerden F.R., Fouché G. (2010). Antimalarial compounds from *Schefflera umbellifera*. S. Afr. J. Bot..

[39] Wattanapiromsakul C., Chanthathamrongsiri N., Bussarawit S., Yuenyongsawad S., Plubrukarn A., Suwanborirux K. (2009). 8-Isocyanoamphilecta-11(20), 15-diene, a new antimalarial isonitrile diterpene from the sponge *Ciocalapata* sp. Can. J. Chem..

[40] Wright A.D., Lang-Unnasch N. (2009). Diterpene formamides from the tropical marine sponge *Cymbastela hooperi* and their antimalarial activity *in vitro*. J. Nat. Prod..

[41] Herath H.M.T.B., Herath W.H.M.W., Carvalho P., Khan S.I., Tekwani B.L., Duke S.O., Tomaso-Peterson M., Nanayakkara N.P.D. (2009). Biologically active tetranorditerpenoids from the fungus *Sclerotinia homoeocarpa* causal agent of dollar spot in turfgrass. J. Nat. Prod..

[42] Dettrakul S., Surerum S., Rajviroongit S., Kittakoop P. (2009). Biomimetic transformation and biological activities of globiferin, a terpenoid benzoquinone from *Cordia globifera*. J. Nat. Prod..

[43] Rao T.S.P., Sarma N.S., Murthy Y.L.N., Kantamreddi V., Wright C.W., Parameswaran P.S. (2010). New polyhydroxy sterols from the marine sponge *Callyspongia fibrosa* (Ridley & Dendly). Tetrahedron Lett..

[44] Oshimi S., Takasaki A., Hirasawa Y., Hosoya T., Awang K., Hadi A.H.A., Ekasari W., Widyawaruyanti A., Morita H. (2009). Delaumonones A and B, new antiplasmodial quassinoids from *Laumoniera bruceadelpha*. Chem. Pharm. Bull..

[45] Houël E., Bertani S., Bourdy G., Deharo E., Jullian V., Valentin A., Chevalley S., Stien D. (2009). Quassinoid constituents of *Quassia amara* L. leaf herbal tea. Impact on its antimalarial activity and cytotoxicity. J. Ethnopharmacol..

[46] Cachet N., Hoakwie F., Bertani S., Bourdy G., Deharo E., Stien D., Houel E., Gornitzka H., Fillaux J., Chevalley S., Valentin A., Jullian V. (2009). Antimalarial activity of simalikalactone E, a new quassinoid from *Quassia amara* L. (Simaroubaceae). Antimicrob. Agents Chemother..

[47] Chianese G., Yerbanga S.R., Lucantoni L., Habluetzel A., Basilico N., Taramelli D., Fattorusso E., Taglialatela-Scafati O. (2010). Antiplasmodial triterpenoids from the fruits of Neem, *Azadirachta indica*. J. Nat. Prod..

[48] Ramalhete C., Lopes D., Mulhovo S., Molnár J., Rosário V.E., Ferreira M.J.U. (2010). New antimalarials with a triterpenic scaffold from *Momordica balsamina*. Bioorg. Med. Chem..

[49] Adams M., Christen M., Plitzko I., Zimmermann S., Brun R., Kaiser M., Hamburger M. (2010). Antiplasmodial lanostanes from the *Ganoderma lucidum* mushroom. J. Nat. Prod..

[50] Elfita E., Muharni M., Latief M., Darwati D., Widiyantoro A., Supriyatna S., Bahti H.H., Dachriyanus D., Cos P., Maes L., Foubert K., Apers S., Pieters L. (2009). Antiplasmodial and other constituents from four Indonesian *Garcinia spp*. Phytochemistry.

[51] Isaka M., Yangchum A., Rachtawee P., Komwijit S., Lutthisungneon A. (2010). Hopane-type triterpenes and binaphthopyrones from the scale insect pathogenic fungus *Aschersonia paraphysata* BCC 11964. J. Nat. Prod..

[52] Maregesi S.M., Hermans N., Dhooghe L., Cimanga K., Ferreira D., Pannecouque C., Vanden Berghe D.A., Cos P., Maes L., Vlietinck A.J., Apers S., Pieters L. (2010). Phytochemical and biological investigations of *Elaeodendron schlechteranum*. J. Ethnopharmacol..

[53] Sá M.S. de, Costa J.F.O., Krettli A.U., Zalis M.G., Maia G.L. de A., Sette I.M.F., Câmara C. de A., Barbosa-Filho J.M., Giulietti-Harley A.M., dos Santos R.R., Soares M.B.P. (2009). Antimalarial activity of betulinic acid and derivatives *in vitro* against *Plasmodium falciparum* and *in vivo* in *P. berghei*-infected mice. Parasitol. Res..

[54] Santos D.A.P., Braga P.A.C., Silva M.F.G.F., Fernandes J.B., Vieira P.C., Magalhães A.F., Magalhães E.G., Marsaioli A.J., Moraes V.R.S., Rattray L., Croft S.L. (2009). Anti-African trypanocidal and antimalarial activity of natural flavonoids, dibenzoylmethanes and synthetic analogues. J. Pharm. Pharmacol..

[55] Agnihotri V.K., ElSohly H.N., Smillie T.J., Khan I.A., Walker L.A. (2009). Constituents of *Leonotis leonurus* flowering tops. Phytochemistry Lett..

[56] Adams M., Plitzko I., Kaiser M., Brun R., Hamburger M. (2009). HPLC-profiling for antiplasmodial compounds 3-Methoxycarpachromene from *Pistacia atlantica*. Phytochemistry Lett..

[57] Songsiang U., Wanich S., Pitchuanchom S., Netsopa S., Uanporn K., Yenjai C. (2009). Bioactive constituents from the stems of *Dalbergia parviflora*. Fitoterapia.

[58] Froelich S., Schubert C., Jenett-Siems K., Preedy V. R. (2009). Antimalarials from prenylated chalcone derivatives of hops. Beer in Health and Disease Prevention.

[59] Cimanga R.K., Tona G.L., Kambu O.K., Mesia G.K., Muyembe J.J.T., Apers S., Totte J., Pieters L., Vlietinck A.J. (2009). Antimalarial, antiamoebic and cytotoxic activities of some extracts and isolated constituents from the leaves of *Morinda morindoides* (Baker) Milne-Redh. (Rubiaceae). Recent Prog. Med. Plants.

[60] Dhooghe L., Maregesi S., Mincheva I., Ferreira D., Marais J.P.J., Lemière F., Matheeussen A., Cos P., Maes L., Vlietinck A., Apers S., Pieters L. (2010). Antiplasmodial activity of (I-3,II-3)-biflavonoids and other constituents from *Ormocarpum kirkii*. Phytochemistry.

[61] Ekasari W., Widyawaruyanti A., Zaini N.C., Syafruddin D., Honda T., Morita H. (2009). Antimalarial activity of Cassiarin a from the leaves of *Cassia siamea*. Heterocycles.

[62] Oshimi S., Deguchi J., Hirasawa Y., Ekasari W., Widyawaruyanti A., Wahyuni T.S., Zaini N.C., Shirota O., Morita H. (2009). Cassiarins C-E, antiplasmodial alkaloids from the flowers of *Cassia siamea*. J. Nat. Prod..

[63] Tchinda A.T., Fuendjiep V., Sajjad A., Matchawe C., Wafo P., Khan S., Tane P., Choudhary M.I. (2009). Bioactive compounds from the fruits of *Zanthoxylum leprieurii*. Pharmacologyonline.

[64] Fernandez L.S., Buchanan M.S., Carroll A.R., Feng Y.J., Quinn R.J., Avery V.M. (2009). Flinderoles A-C: Antimalarial bis-indole alkaloids from *Flindersia* species. Org. Lett..

[65] Fernandez L.S., Sykes M.L., Andrews K.T., Avery V.M. (2010). Antiparasitic activity of alkaloids from plant species of Papua New Guinea and Australia. Int. J. Antimicrob. Agents.

[66] Lopes S.C.P., Blanco Y.C., Justo G.Z., Nogueira P.A., Rodrigues F.L.S., Goelnitz U., Wunderlich G., Facchini G., Brocchi M., Duran N., Costa F.T.M. (2009). Violacein extracted from *Chromobacterium violaceum* inhibits *Plasmodium* growth *in vitro* and *in vivo*. Antimicrob. Agents Chemother..

[67] Buchanan M.S., Davis R.A., Duffy S., Avery V.M., Quinn R.J. (2009). Antimalarial benzylisoquinoline alkaloid from the rainforest tree *Doryphora sassafras*. J. Nat. Prod..

[68] Wangchuk P., Bremner J.B., Samten, Rattanajak R., Kamchonwongpaisan S. (2010). Antiplasmodial agents from the Bhutanese medicinal plant *Corydalis calliantha*. Phytother. Res..

[69] Thongthoom T., Songsiang U., Phaosiri C., Yenjai C. (2010). Biological activity of chemical constituents from *Clausena harmandiana*. Arch. Pharmacal. Res..

[70] Wansi J.D., Hussain H., Tcho A.T., Kouam S.F., Specht S., Sarite S.R., Hoerauf A., Krohn K. (2010). Antiplasmodial activities of furoquinoline alkaloids from *Teclea afzelii*. Phytother. Res..

[71] Mueller D., Davis R.A., Duffy S., Avery V.M., Camp D., Quinn R.J. (2009). Antimalarial activity of azafluorenone alkaloids from the Australian tree *Mitrephora diversifolia*. J. Nat. Prod..

[72] Laville R., Thomas O.P., Berrué F., Marquez D., Vacelet J., Amade P. (2009). Bioactive guanidine alkaloids from two Caribbean marine sponges. J. Nat. Prod..

[73] Yamada M., Takahashi Y., Kubota T., Fromont J., Ishiyama A., Otoguro K., Yamada H., Omura S., Kobayashi J. (2009). Zamamidine C, 3,4-dihydro-6-hydroxy-10,11-epoxymanzamine A, and 3,4-dihydromanzamine J N-oxide, new manzamine alkaloids from sponge *Amphimedon sp*. Tetrahedron.

[74] Samoylenko V., Khan S.I., Jacob M.R., Tekwani B.L., Walker L.A., Hufford C.D., Muhammad I. (2009). Muhammad, I. Bioactive (+)-manzamine A and (+)-8-hydroxymanzamine A tertiary bases and salts from *Acanthostrongylophora ingens* and their preparations. Nat. Prod. Commun..

[75] Wangchuk P., Bremner J.B., Samten, Skelton B.W., White A.H., Rattanajak R., Kamchonwongpaisan S. (2010). Antiplasmodial activity of atisinium chloride from the Bhutanese medicinal plant, *Aconitum orochryseum*. J. Ethnopharmacol..

[76] Chen Y., Li S.Y., Sun F., Han H., Zhang X., Fan Y.Y., Tai G.H., Zhou Y.F. (2010). *In vivo* antimalarial activities of glycoalkaloids isolated from Solanaceae plants. Pharm. Biol..

[77] Kumarihamy M., Fronczek F.R., Ferreira D., Jacob M., Khan S.I., Nanayakkara N.P.D. (2010). Bioactive 1,4-dihydroxy-5-phenyl-2-pyridinone alkaloids from *Septoria pistaciarum*. J. Nat. Prod..

[78] Na M., Ding Y., Wang B., Tekwani B.L., Schinazi R.F., Franzblau S., Kelly M., Stone R., Li X.-C., Ferreira D., Hamann M.T. (2010). Anti-infective discorhabdins from a deep-water Alaskan sponge of the genus *Latrunculia*. J. Nat. Prod..

[79] Yang X.Z., Davis R.A., Buchanan M.S., Duffy S., Avery V.M., Camp D., Quinn R.J. (2010). Antimalarial bromotyrosine derivatives from the Australian marine sponge *Hyattella sp*. J. Nat. Prod..

[80] Davis R.A., Carroll A.R., Andrews K.T., Boyle G.M., Tran T.L., Healy P.C., Kalaitzis J.A., Shivas R.G. (2010). Pestalactams A-C: novel caprolactams from the endophytic fungus *Pestalotiopsis sp*. Org. Biomol. Chem..

[81] Davis R.A., Duffy S., Avery V.M., Camp D., Hooper J.N.A., Quinn R.J. (2010). (+)-7-Bromotrypargine: an antimalarial β-carboline from the Australian marine sponge *Ancorina sp*. Tetrahedron Lett..

[82] Scala F., Fattorusso E., Menna M., Taglialatela-Scafati O., Tierney M., Kaiser M., Tasdemir D. (2010). Bromopyrrole alkaloids as lead compounds against protozoan parasites. Mar. Drugs.

[83] Linington R.G., Clark B.R., Trimble E.E., Almanza A., Ureña L.D., Kyle D.E., Gerwick W.H. (2009). Antimalarial peptides from marine cyanobacteria: Isolation and structural elucidation of gallinamide A. J. Nat. Prod..

[84] Guerrant W., Mwakwari S.C., Chen P.C., Khan S.I., Tekwani B.L., Oyelere A.K. (2010). A structure-activity relationship study of the antimalarial and antileishmanial activities of nonpeptide macrocyclic histone deacetylase inhibitors. ChemMedChem.

[85] Haritakun R., Rachtawee P., Chanthaket R., Boonyuen N., Isaka M. (2010). Butyrolactones from the fungus *Aspergillus terreus* BCC 4651. Chem. Pharm. Bull..

[86] Lanfranchi D.A., Laouer H., El Kolli M., Prado S., Maulay-Bailly C., Baldovini N. (2010). Bioactive phenylpropanoids from *Daucus crinitus* Desf. from Algeria. J. Agric. Food Chem..

[87] Gachet M.S., Kunert O., Kaiser M., Brun R., Muñoz R.A., Bauer R., Schühly W. (2010). Jacaranone-derived glucosidic esters from *Jacaranda glabra* and their activity against *Plasmodium falciparum*. J. Nat. Prod..

[88] Kaou A.M., Mahiou-Leddet V., Canlet C., Debrauwer L., Hutter S., Laget M., Faure R., Azas N., Ollivier E. (2010). Antimalarial compounds from the aerial parts of *Flacourtia indica* (Flacourtiaceae). J. Ethnopharmacol..

[89] Tangmouo J.G., Ho R., Matheeussen A., Lannang A.M., Komguem J., Messi B.B., Maes L., Hostettmann K. (2010). Antimalarial activity of extract and norbergenin derivatives from the stem bark of *Diospyros sanza-minika* A. Chevalier (Ebenaceae). Phytother. Res..

[90] Dhooghe L., Maregesi S., Maes L., Cos P., Apers S., Vlietinck A., Pieters L. (2009). Bioassay guided isolation of antiplasmodial constituents from *Ormocarpum kirkii*. Planta Med..

[91] Laphookhieo S., Maneerat W., Koysomboon S. (2009). Antimalarial and cytotoxic phenolic compounds from *Cratoxylum maingayi* and *Cratoxylum cochinchinense*. Molecules.

[92] Hou Y., Cao S., Brodie P.J., Callmander M.W., Ratovoson F., Rakotobe E.A., Rasamison V.E., Ratsimbason M., Alumasa J.N., Roepe P.D., Kingston D.G.I. (2009). Antiproliferative and antimalarial anthraquinones of *Scutia myrtina* from the Madagascar forest. Bioorg. Med. Chem..

[93] Fattorusso C., Persico M., Calcinai B., Cerrano C., Parapini S., Taramelli D., Novellino E., Romano A., Scala F., Fattorusso E., Taglialatela-Scafati O. (2010). Manadoperoxides A-D from the Indonesian sponge *Plakortis cfr. simplex*. Further insights on the structure-activity relationships of simple 1,2-dioxane antimalarials. J. Nat. Prod..

[94] Mohammed R., Peng J., Kelly M., Yousaf M., Winn E., Odde S., Bie Z., Xie A., Doerksen R.J., Hamann M.T. (2010). Polyketide-peroxides from a species of Jamaican *Plakortis* (Porifera: Demospongiae). Aust. J. Chem..

[95] Jiménez-Romero C., Ortiz I., Vicente J., Vera B., Rodríguez A.D., Nam S., Jove R. (2010). Bioactive cycloperoxides isolated from the Puerto Rican sponge *Plakortis halichondrioides*. J. Nat. Prod..

[96] Ueoka R., Nakao Y., Kawatsu S., Yaegashi J., Matsumoto Y., Matsunaga S., Furihata K., van Soest R.W.M., Fusetani N. (2009). Gracilioethers A−C, antimalarial metabolites from the marine sponge *Agelas gracilis*. J. Org. Chem..

[97] Gutiérrez M., Tidgewell K., Capson T.L., Engene N., Almanza A., Schemies J., Jung M., Gerwick W.H. (2010). Malyngolide dimer, a bioactive symmetric cyclodepside from the Panamanian marine cyanobacterium *Lyngbya majuscula*. J. Nat. Prod..

[98] Kumari P., Misra K., Sisodia B.S., Faridi U., Srivastava S., Luqman S., Darokar M.P., Negi A.S., Gupta M.M., Singh S.C., Kumar J.K. (2009). A promising anticancer and antimalarial component from the leaves of *Bidens pilosa*. Planta Med..

[99] Tobinaga S., Sharma M.K., Aalbersberg W.G.L., Watanabe K., Iguchi K., Narui K., Sasatsu M., Waki S. (2009). Isolation and identification of a potent antimalarial and antibacterial polyacetylene from *Bidens pilosa*. Planta Med..

[100] Gupta P., Vasudeva N. (2010). *In vitro* antiplasmodial and antimicrobial potential of *Tagetes erecta* roots. Pharm. Biol..

[101] Xu L., He Z., Xue J., Chen X., Wei X. (2010). Resorcylic acid lactones from a *Paecilomyces* fungus. J. Nat. Prod..

[102] Khumkomkhet P., Kanokmedhakul S., Kanokmedhakul K., Hahnvajanawong C., Soytong K. (2009). Antimalarial and cytotoxic depsidones from the fungus *Chaetomium brasiliense*. J. Nat. Prod..

[103] Marti G., Eparvier V., Moretti C., Susplugas S., Prado S., Grellier P., Retailleau P., Guéritte F., Litaudon M. (2009). Antiplasmodial benzophenones from the trunk latex of *Moronobea coccinea* (Clusiaceae). Phytochemistry.

[104] Marti G., Eparvier V., Moretti C., Prado S., Grellier P., Hue N., Thoison O., Delpech B., Guéritte F., Litaudon M. (2010). Antiplasmodial benzophenone derivatives from the root barks of *Symphonia globulifera* (Clusiaceae). Phytochemistry.

[105] Lane A.L., Stout E.P., Lin A.S., Prudhomme J., Le Roch K., Fairchild C.R., Franzblau S.G., Hay M.E., Aalbersberg W., Kubanek J. (2009). Antimalarial bromophycolides J-Q from the Fijian red alga *Callophycus serratus*. J. Org. Chem..

[106] Kornsakulkarn J., Thongpanchang C., Chainoy R., Choowong W., Nithithanasilp S., Thongpanchang T. (2010). Bioactive metabolites from cultures of basidiomycete *Favolaschia tonkinensis*. J. Nat. Prod..

[107] Bunyapaiboonsri T., Yoiprommarat S., Intereya K., Rachtawee P., Hywel-Jones N.L., Isaka M. (2009). Isariotins E and F, spirocyclic and bicyclic hemiacetals from the entomopathogenic fungus *Isaria tenuipes* BCC 12625. J. Nat. Prod..

[108] Lebouvier N., Jullian V., Desvignes I., Maurel S., Parenty A., Dorin-Semblat D., Doerig C., Sauvain M., Laurent D. (2009). Antiplasmodial activities of homogentisic acid derivative protein kinase inhibitors isolated from a *Vanuatu* marine sponge* Pseudoceratina sp*. Mar. Drugs.

[109] Moosophon P., Kanokmedhakul S., Kanokmedhakul K., Soytong K. (2009). Prenylxanthones and a bicyclo [3.3.1]nona-2,6-diene derivative from the fungus *Emericella rugulosa*. J. Nat. Prod..

[110] Chung I.M., Seo S.H., Kang E.Y., Park W.H., Moon H.I. (2009). Anti-malarial activity of 6-(8'Z-pentadecenyl)-salicylic acid from *Viola websteri* in mice. Malar. J..

[111] Rangkaew N., Suttisri R., Moriyasu M., Kawanishi K. (2009). A new acyclic diterpene acid and bioactive compounds from *Knema glauca*. Arch. Pharm. Res..

